# A FoxM1/Smad4 positive feedback loop promotes pancreatic cancer progression

**DOI:** 10.1038/s41419-026-08697-y

**Published:** 2026-04-10

**Authors:** Banzhan Ruan, Bingshu Wang, Xiaodian Zhang, Fujin Liu, Zhenling Wan, Yan Chen, Julan Wu, Chun Luo, Wenyan Lu, Yanda Lu, Shaojiang Zheng

**Affiliations:** 1 Key Laboratory of Emergency and Trauma of Ministry of Education, Engineering Research Center for Hainan Biological Sample Resources of Major Diseases, Hainan Branch of National Clinical Research Center for Cancer, the First Affiliated Hospital, Hainan Medical University, Haikou, China; 2 Department of Biology, School of Basic Medicine, Hainan Medical University, Haikou, China; 3 Department of Pathology, The Second Affiliated Hospital of Hainan Medical University, Haikou, China; 4 Department of Pathology, Hainan General Hospital, Hainan Medical University, Haikou, China; 5 Department of Pathology, Hainan Women and Children’s Medical Center, Hainan Medical University, Haikou, China; 6 Hainan Branch of State Key Laboratory of Systems Medicine for Cancer, Medical Academy of Hainan Province, Hainan Medical University, Haikou, China

**Keywords:** Oncogenes, Mechanisms of disease

## Abstract

Pancreatic cancer is a highly lethal disease characterized by rapid onset, aggressive progression, and limited treatment options. The involvement of FoxM1 in the TGF-β/Smad signaling pathway has been linked to pancreatic cancer progression; however, the mechanisms behind the cooperative regulation of TGF-β signaling by FoxM1 and Smad4 remain poorly understood. In this study, we utilized molecular cytology techniques, animal models, and human pancreatic cancer tissues to investigate the role of FoxM1 in Smad4 stabilization and its regulation of TGF-β signaling. Our findings reveal that FoxM1 inhibits ubiquitin-proteasome-mediated degradation of Smad4, resulting in its stabilization. Once translocated into the nucleus, Smad4 binds to the FoxM1 promoter region, inducing FoxM1 expression and forming a positive feedback loop. Furthermore, we observed significantly higher expression of this feedback loop in pancreatic cancer tissues compared to adjacent normal tissues, with markedly elevated levels in poorly differentiated tissues compared to well-differentiated ones. Therefore, the loop aberrantly activates the TGF-β pathway, driving pancreatic cancer progression. These findings uncover a novel mechanism of TGF-β pathway activation and provide potential new targets for the prevention and treatment of pancreatic cancer.

**Figure:**
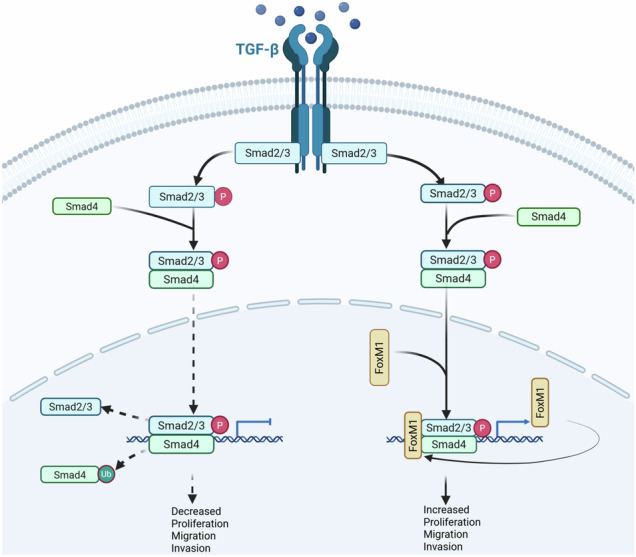
This study elucidates that FoxM1 functions to impede the ubiquitin proteasome-mediated degradation of Smad4, consequently stabilizing it. Following nuclear translocation, Smad4 binds to the FoxM1 promoter region, initiating FoxM1 expression and establishing a positive feedback loop. This loop plays a pivotal role in promoting pancreatic cancer development and migration by aberrantly activating the TGF-β pathway.

This study elucidates that FoxM1 functions to impede the ubiquitin proteasome-mediated degradation of Smad4, consequently stabilizing it. Following nuclear translocation, Smad4 binds to the FoxM1 promoter region, initiating FoxM1 expression and establishing a positive feedback loop. This loop plays a pivotal role in promoting pancreatic cancer development and migration by aberrantly activating the TGF-β pathway.

## Introduction

Pancreatic cancer is a highly aggressive malignancy characterized by its insidious onset, rapid progression, and limited treatment options, contributing to an exceptionally poor prognosis [1, 2]. According to global cancer statistics, both the incidence and mortality of pancreatic cancer are rising worldwide, with particularly notable increases observed in developing countries [3]. These alarming trends underscore the urgent need to identify novel therapeutic targets to combat this devastating disease [4]. As such, the search for new molecular drivers of pancreatic cancer has become a key objective in translational oncology research.

Among the pathways implicated in pancreatic tumorigenesis, the canonical transforming growth factor-beta (TGF-β)/Smad signaling pathway plays a pivotal role in both the initiation and progression of various cancers, including pancreatic cancer [5, 6]. This pathway is activated when TGF-β ligands bind to type II TGF-β receptors (TβRII) on the cell surface, leading to the phosphorylation and activation of type I receptors (TβRI). In turn, TβRI activates Smad2 and Smad3 in the cytoplasm, which then form a complex with Smad4. This complex translocated into the nucleus, where it regulated the transcription of target genes involved in tumor progression and metastasis [5].

Recent studies have highlighted the involvement of Forkhead box M1 (FoxM1), a transcription factor known for its role in cell cycle regulation and oncogenesis, in modulating the TGF-β/Smad pathway [7–9]. For instance, FoxM1 has been shown to promote ovarian cancer invasion and metastasis by transcriptionally up-regulating *DLX1* through activation of the TGF-β/Smad4 signaling [10]. Conversely, suppression of FoxM1 can inhibit the Smad2/3 signaling axis and attenuate processes such as cardiac vascular endothelial-mesenchymal transition and migration [9]. In our previous work, we demonstrated that FoxM1 promotes breast cancer metastasis by activating the TGF-β/Smad3 pathway, in part through competition with Smad3 for TIF1γ binding and by stabilizing Smad4 through inhibition of its ubiquitin-mediated degradation [11]. These results suggest that FoxM1 may enhance TGF-β/Smad signaling via post-translational mechanisms. However, the mechanisms by which FoxM1 and Smad4 cooperate to promote pancreatic cancer progression remain to be elucidated.

In this study, we aim to systematically investigate the role of the FoxM1/Smad4 positive feedback signaling loop in the progression of pancreatic cancer, using a combination of molecular cytology techniques and experimental animal models both in vitro and in vivo. We will explore the underlying molecular mechanism driving this feedback loop and evaluate its clinical significance through analysis of human pancreatic cancer specimens. The findings from this work are expected to provide new mechanistic insights and identify potential therapeutic targets for the prevention and treatment of pancreatic cancer.

## Materials and methods

### Cell culture

HPDE6-C7 was acquired from Beina Chuanglian Biotech (Suzhou, China), while Panc-1, SW1990, Panc10.05, CFPAC-1, PaTu8988 (PaTu8988-T), MIA-PACA-2, Capan-1, Capan-2, BxPC3, and AsPC-1 were obtained from KeyGEN Biotech (Nanjing, China). The cell lines were cultured in various media depending on their requirements. Specifically, SW1990 was grown in L-15 medium with 10% FBS, CFPAC-1 and Capan-1 in IMDM medium, Capan-2 in McCoy’s 5 A, PaTu8988 using RPMI-160 medium, and the other cell lines were cultured in DMEM medium. Except for Panc10.05 and MIA-PACA-2, which were grown in 85% RPMI-1640 + 15% FBS + 10 μg/ml insulin and 87.5% DMEM + 10% FBS + 2.5% horse serum, respectively, all other eight cell lines were cultured in DMEM medium with 10% FBS. All cell lines were incubated at 37 °C with 5% CO_2_, authenticated and routinely tested for Mycoplasma contamination using the MycoGuard™ Mycoplasma PCR Detection Kit 2.0 (iGeneBio, MP004).

### Antibodies and chemical reagents

The primary antibodies used in this research were as follows: anti-GAPDH (KeyGEN Biotech, KGAA002, WB 1:5000), anti-FoxM1 (Abcam, ab207298, WB 1:1000, IF 1:250, IP 1:100), anti-Smad4 (Santa Cruz, sc-7966, WB 1:500, IF 1:100), anti-Smad4 (Abcam, ab236321, IP 1:100), anti-Ub (Abcam, ab134953, WB 1:1000), anti-E-cadherin (Abcam, ab40772, WB 1:2000), anti-N-cadherin (Abcam, ab98952, WB 1:1000), anti-p-Smad2 (Abcam, ab188334, WB 1:5000), anti-p-Smad3 (Abcam, ab52903, WB 1:2000), anti-Smad2 (Abcam, ab40855, WB 1:5000), anti-Smad3 (Abcam, ab40854, WB 1:5000), anti-Snail1 (Abcam, ab216347, WB 1:1000), anti-Snail2 (Abcam, ab51772, WB 1:1000), anti-Twist (Abcam, ab175430, WB 1:1000), anti-Vimentin (Abcam, ab92547, WB 1:3000), anti-Zeb1 (Abcam, ab203829, WB 1:1000), anti-Zeb2 (Abcam, ab138222, WB 1:1000), and anti-ZO-1 (Abcam, ab96587, WB 1:1000). The secondary antibodies included HRP-linked anti-Rabbit IgG (KeyGEN Biotech, KGAA35, WB 1:5000), HRP-linked anti-Mouse IgG (KeyGEN Biotech, KGAA37, WB 1:5000), and Fluorescein-conjugated anti-Rabbit IgG (KeyGEN Biotech, KGAA99, IF 1:500).

The chemical reagents utilized in this study were as follows: recombinant human TGF-β1 protein (SinoBiological, 10804-H08H) as a cytokine, MG132 (MCE, HY-13259), chloroquine (MCE, HY-17598A), and cycloheximide (Aladdin, C112766).

### Patient samples

Pancreatic cancer tissue microarray (TMA) chips (HPanA170Su04) used in this study were purchased from Shanghai Outdo Biotech Co., Ltd., consisting of 170 cores, including 90 patients. Tumor and paracancerous tissues were freshly excised from 7 poorly differentiated and 9 well-differentiated pancreatic cancer patients undergoing surgical resection at Hainan Medical University Hainan Hospital.

### Migration, invasion and colony formation assays

To determine cell migration, invasion and colony formation, Patu8988 cells or Panc10.05 cells in the exponential growth phase were seeded in 6-well plates. After cell adhesion, the medium was replaced with a drug-containing medium. A negative control group was included. After 48 h of drug treatment, cells were digested with 0.25% trypsin (without EDTA) and collected for subsequent determination of cell migration and invasion.

For cell migration determination, an equal number of cells from each group was seeded in a 6-well plate. The following day, when cells reached 80% confluence, a sterile pipette tip was used to scratch the cells in the plate, which were then washed with PBS. After culturing for 24 h in fresh culture medium with 1% FBS, the cell scratch width was measured by taking pictures under a microscope.

For cell invasion determination, Matrigel (BD Biosciences, Bedford, USA) was diluted with incomplete medium, and 30 µL was added to the upper chamber. After incubation at 37 °C for 120 min to form Matrigel, cells were seeded into the upper chamber. An equal number of cells from each group were cultured in serum-free medium for 24 h, adjusted to a cell density of 1 × 10^5^ cells/mL, and 100 µL of the cell suspension was added to the Transwell inserts. Meanwhile, 500 µL of medium containing 20% FBS was added to the lower chamber. After 24 h of incubation at 37 °C, uninvaded cells in the upper inserts were removed with a cotton swab. Invaded cells in the lower chamber were fixed with 4% paraformaldehyde and stained with 0.1% crystal violet. The number of invaded cells was counted under a 200× magnification microscope. Three visual fields were randomly selected in each group, and the experiment was repeated three times independently.

For the colony formation assay, 500 cells were seeded in 6-well plates in triplicate and cultured with TGF-β1 or vehicle for 12 days at 37 °C. For FoxM1 knockdown, cells were transfected with siRNA 24 h prior to seeding. After incubation, colonies were fixed with 4% paraformaldehyde, stained with 0.1% crystal violet, rinsed thoroughly with water, air-dried, and counted.

### Vectors and mRNA expression measurement

Three small interference RNAs (siRNAs) were used for transient FoxM1 silencing in pancreatic tumor cells: siRNA1 (5’-CCAACAGGAGUCUAAUCAA-3’), siRNA2 (5’-CAAUCGUUCUCUGACAGAA-3’) and siRNA3 (5’-GGAAGCGCAUGACUUUGAA-3’). For stable knockdown, short hairpin RNA (shRNA) lentiviral constructs targeting three human FOXM1 sites-shRNA1 (5’-GCCCAACAGGAGTCTAATCAA-3’), shRNA2 (5’-GCCAATCGTTCTCTGACAGAA-3’), and shRNA3 (5’-CGCCGGAACATGACCATCAAA-3’)-were generated. For the overexpression lentiviral vector, the FoxM1 coding sequence was cloned into a lentiviral expression vector containing CMV-MCS-Luc2-IRES-Puromycin. All vectors were sequenced to confirm their accuracy before virus packaging and transfection.

After transfection, mRNA expression levels were measured using Real-time qPCR. Total RNA was extracted from pancreatic cancer cells using TRIzol, and 1 μg of RNA from each sample was used to synthesize cDNA with PrimeScript RT Master Mix (TaKaRa, RR036B). cDNA was used in a Real-time qPCR assay with the One Step PrimeScript RT-PCR Kit (TaKaRa, RR086B). The primers used in this study were FOXM1-rt-F 5’-GGTTTTCTCCTTTGCTTCCAGT-3’, FOXM1-rt-R 5’- CTTTGATGGGTCTCGCTAAGTG-3’, and GAPDH-rt-F 5’- CAAATTCCATGGCACCGTCA-3’, GAPDH-rt-R 5’- AGCATCGCCCCACTTGATTT-3’. mRNA expression was quantified relative to GAPDH using the 2^-ΔΔCT^ method.

### Immunoblotting and co-immunoprecipitation

Protein samples were extracted using the Total Protein Extraction Kit (KeyGen BioTECH, KGB5303) and quantified by the BCA method for Western blot analysis. Equal amounts of protein were loaded onto SDS-PAGE gels and separated using the Glycine–Tris buffer system (KeyGen BioTECH, KGC4101). The separated proteins were transferred onto nitrocellulose membranes under constant current and probed with appropriate primary and secondary antibodies for target detection.

Co-immunoprecipitation (co-IP) was performed according to our recent publication [12]. Briefly, cells were lysed using the WB/IP Lysis Buffer Kit (KeyGen BioTECH, KGB5202). The clarified lysates were incubated with the indicated primary antibody or control IgG overnight at 4 °C with gentle rotation, followed by incubation with Protein G Agarose for 3 h at 4 °C. The agarose beads were washed with PBS, and the immunoprecipitated proteins were eluted by boiling in 1x SDS-PAGE loading buffer. The eluted proteins were then subjected to SDS-PAGE and immunoblotting as described above.

### Immunostaining and multiplex immunofluorescence staining

Immunostaining was performed as described [13]. Briefly, cells on slides were fixed in 4% paraformaldehyde for 10 min, and then permeabilized using 0.5% Triton X-100 for 3 min. The cells were then subjected to immunostaining with specific antibodies. Nuclei were counterstained with DAPI, and slides were mounted with coverslips using Prolong Gold Antifade reagent. After 24 h of air-drying, images were captured using confocal microscopy at four channels (FITC, TRITC, DAPI, and DIC).

For the multiplex immunofluorescence staining assay, tissue microarrays (TMAs) were heated at 63 °C for 1 h, followed by deparaffinization in xylene (twice, 15 min each) and rehydration through a graded ethanol series (100% for 7 min, then 90%, 80%, and 70% for 5 min each). The slides were then rinsed with Tris-Buffered Saline with Tween 20 (TBST) (three times, 10 min each). Antigen retrieval was performed by immersing the slides in preheated citrate antigen retrieval solution (ZSGB-BIO) at 95 °C for 20 min, followed by cooling to room temperature. After washing with TBST, the tissues were incubated with a blocking buffer for 10 min at room temperature. The blocking buffer was then replaced with the primary antibody anti-FoxM1 (Abcam, ab207298, IF 1:500), and the slides were incubated at room temperature for 1 h. The primary antibody was washed off with TBST (three times, 10 min each), and the secondary antibody Opal 520 (PerkinElmer, NEL801001KT, 1:100) was applied for 10 min at room temperature. After washing with TBST, residual primary and secondary antibodies were removed by heating. Subsequent targets were stained sequentially, following the same steps from antigen retrieval to secondary antibody removal as described for FoxM1. The additional primary antibodies used were anti-Smad4 (Abcam, ab40759, 1:50), anti-p-Smad3 (Abcam, ab52903, 1:50), and anti-CK7 (Abcam, ab181598, 1:10), with their respective fluorescent secondary antibodies Opal 570, Opal 650, and Opal 690 (PerkinElmer, NEL801001KT, 1:100). After staining for all four targets, cell nuclei were counterstained with DAPI for 10 min at room temperature. Finally, the slides were mounted using VECTASHIELD HardSet Antifade Mounting Medium (Vector Labs, H-1400).

### Luciferase assay

Luciferase can be applied for measuring the activity of promoters, or/and/or the transcription efficiency. A 2000 bp wild-type FoxM1 promoter sequence was synthesized and inserted into the GV238 plasmid (Genechem, Shanghai, China), with the empty GV238-basic plasmid as the control plasmid. BxPC3 cells (3 × 10^4^/well) were co-transfected with 450 ng luciferase reporter plasmid, 50 ng FoxM1 promoter-Renilla-Luciferase plasmid and 50 ng Smad4 expression vector using Lipofectamine 3000(L3000001, Thermo Fisher Scientific, Waltham, Massachusetts, USA). Twenty-four and forty-eight h after transfection, luciferase activities (Firefly and Renilla luciferase) were measured with a Dual-Luciferase assay kit according to the manufacturer’s instructions (Promega, E1910). The results were expressed as the relative firefly luciferase activity ratio to Renilla luciferase activity.

### Electrophoretic mobility shift assay (EMSA)

Nucleoprotein and cytoplasmic protein fractions were isolated using the Nucleoprotein and Cytoplasmic Protein Extraction Kit (KGP150, Jiangsu KGI Biotechnology Co., Ltd., China). Electrophoretic mobility shift assay (EMSA) reactions were prepared according to the manufacturer’s instructions (KGS101, Jiangsu KGI Biotechnology Co., Ltd., China). Biotin-labeled oligonucleotides (biotin probe), biotin-labeled mutant oligonucleotides (mutant probe) and unlabeled oligonucleotides (cold probe) specific for the promoters of FoxM1 and Smad4 were synthesized.

The probes and their corresponding sequences used for detecting FoxM1 and Smad4 are as follows: FOXM1 probe, GAGCCCAGGGGAAGGAAAGAACCTTGTCTGCCATTGTATCTTCAGGGCCTAGCGGTGCCT; FOXM1 mutant probe, GAGCCCAGGGGAAGGAAAGAACCGAGGTTAGCAATGTATCTTCAGGGCCTAGCGGTGCCT; SMAD4 probe, ATACAGCAATAGGTAACTAATACAGACGGAATAGAAGAAGTTTAGTTATAACAATGTCTC; SMAD4 mutant probe, ATACAGCAATAGGTAACTAATACGTATAAGGCAAAAGAAGTTTAGTTATAACAATGTCTC.

### Orthotopic implantation of pancreatic cancer cells

An orthotopic pancreatic tumor model was established as previously described with minor modifications [14]. Briefly, female nude mice aged 5–7 weeks were randomly assigned to six groups (*n* > 6 per group): Patu8988 cells with shFoxM1-3 or corresponding controls (mock and scramble), and Panc10.05 cells overexpressing FoxM1 or corresponding controls (mock and empty vector). Pancreatic cancer cells were suspended at a concentration of 5 × 10^5^ cells in 50 μL PBS and injected into the tail region of the pancreas. During the surgical procedure, mice were anesthetized with isoflurane inhalation (5% for induction and 2% for maintenance). After injection, the peritoneum and skin were carefully sutured, and iodine was used for sterilization. The mice were allowed to naturally awaken before being put back in their housing cages. They were monitored every 2–3 days for body weight, appearance, and general health status, and were euthanized when tumor burden or physical condition met the humane endpoint criteria. Tumor growth was monitored regularly for several weeks by intravital imaging.

### Statistics

All experiments were conducted at least three times, and results were presented as mean ± SD. Differences between groups were analyzed using the unpaired two-tailed Student’s *t* test, with statistical significance defined as * *p* < 0.05 (significant), **p < 0.01 (highly significant), and ***p < 0.001 (very significant). Statistical analyses were conducted using SPSS 20.0 software (IBM), and graphs were generated with GraphPad Prism 8.3.0.

### Compliance with Ethics Requirements

All experiments involving animals were conducted in strict accordance with the guidelines set by the Chinese Regulations of Laboratory Animals and Laboratory Animal-Requirements of Environment and Housing Facilities, approved by the Experimental Animal Committee of Laboratory Animal Center, Hainan Medical University (Approval number: ICAUC-2023-KYL-214). All procedures involving human participants were conducted in accordance with institutional ethical standards and the Declaration of Helsinki. The study protocol was reviewed and approved by the Ethics Committee of Hainan Medical University, Hainan Hospital. Written informed consent was obtained from all patients prior to tissue collection.

## Results

### FoxM1 promotes TGF-β-medicated migration and invasion of pancreatic cancer cells

To investigate the role of FoxM1 in TGF-β/Smad signaling-mediated biological behaviors of pancreatic cancer cells in vitro, we first screened 10 pancreatic cancer cell lines for endogenous FoxM1 expression. Panc10.05 cells exhibited low FoxM1 expression, while PaTu8988 cells showed a high expression level (Fig. S1A, B). Considering FoxM1 baseline expression, TP53 status, and technical robustness, we established FoxM1-overexpressing Panc10.05 cells and FoxM1-knockdown PaTu8988 cells using gene transfection techniques. Quantitative analysis of FoxM1 mRNA and protein levels confirmed the successful generation of stable overexpression and knockdown cell lines (Figs. S1C–F and S2A–D).

**Fig. 1:**
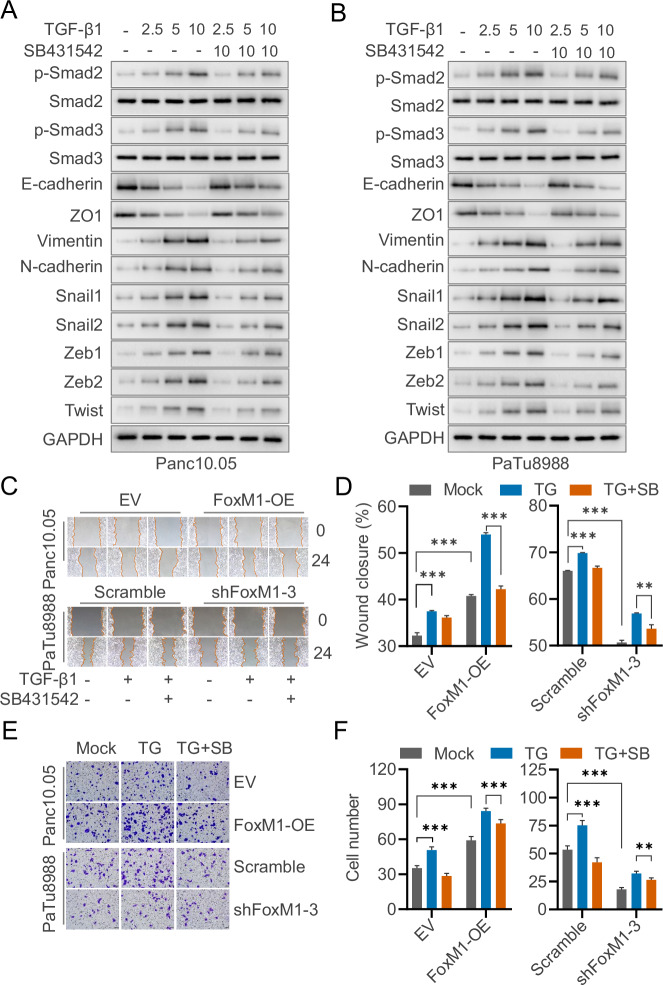
FoxM1 regulates TGF-β-induced migration and invasion of pancreatic cancer cells in vitro. PaTu8988 and Panc10.05 cells were treated with recombinant human cytokine TGF-β1 and its inhibitor SB431542. The expression of the downstream proteins of TGF-β signaling (Smad-2/3 and their phosphorylated) and epithelial-mesenchymal transition-related proteins (N-cadherin, Vimentin, etc.) were measured by Western blotting (**A**, **B**). Scratch and Transwell assays were performed to evaluate cell migration and invasion, respectively. Representative images of the scratch experiment (**C**) and Transwell assay (**E**) are shown, along with quantification of wound closure percentage (**D**) and invaded cells (**F**). TG and SB indicate TGF-β1 and SB431542 treatments, respectively. The data are presented as mean ± SD of triplicate quantification. ns not significant, ** *p* < 0.01, and *** *p* < 0.001.

Next, we evaluated the baseline responsiveness of pancreatic cancer cells, Panc10.05 cells and PaTu8988, to the TGF-β pathway activator TGF-β1 and the inhibitor SB-431542. Immunoblot analysis showed that increasing concentrations of TGF-β1 led to a dose-dependent increase in Smad2/3 phosphorylation, which was partially reversed by SB-431542. In addition, well-characterized downstream effectors of the TGF-β1 signaling pathway—epithelial–mesenchymal transition (EMT)-related proteins such as N-cadherin and Vimentin—were upregulated by TGF-β1 and downregulated upon SB-431542 treatment (Fig. 1A, B). These results confirm that the pancreatic cancer cell lines used in this study are responsive to both TGF-β1 stimulation and SB-431542 inhibition, and that the TGF-β/Smad signaling pathway can be effectively modulated in this system.

To further evaluate the role of FoxM1 in TGF-β1–induced cell motility, we performed wound healing assays. FoxM1 overexpression significantly promoted migration in Panc10.05 cells, whereas FoxM1 depletion inhibited migration in Patu8988 cells. TGF-β1 treatment enhanced the migratory capacity of pancreatic cancer cells, an effect that was further amplified by FoxM1 overexpression. Conversely, the TGF-β receptor inhibitor SB-431542 partially attenuated this enhancement (Figs. 1C, D and S3A, B). Similar results were observed in Transwell invasion assays, where FoxM1 depletion suppressed, but overexpression promoted cell invasiveness in a TGF-β1–dependent manner, and SB-431542 partially reversed these effects (Figs. 1E, F and S3C, D).

Together, these findings demonstrate that FoxM1 promotes pancreatic cancer cell migration and invasion, at least in part, through the TGF-β1 signaling pathway.

### FoxM1 enhances TGF-β-induced pancreatic tumorigenesis

**Fig. 2:**
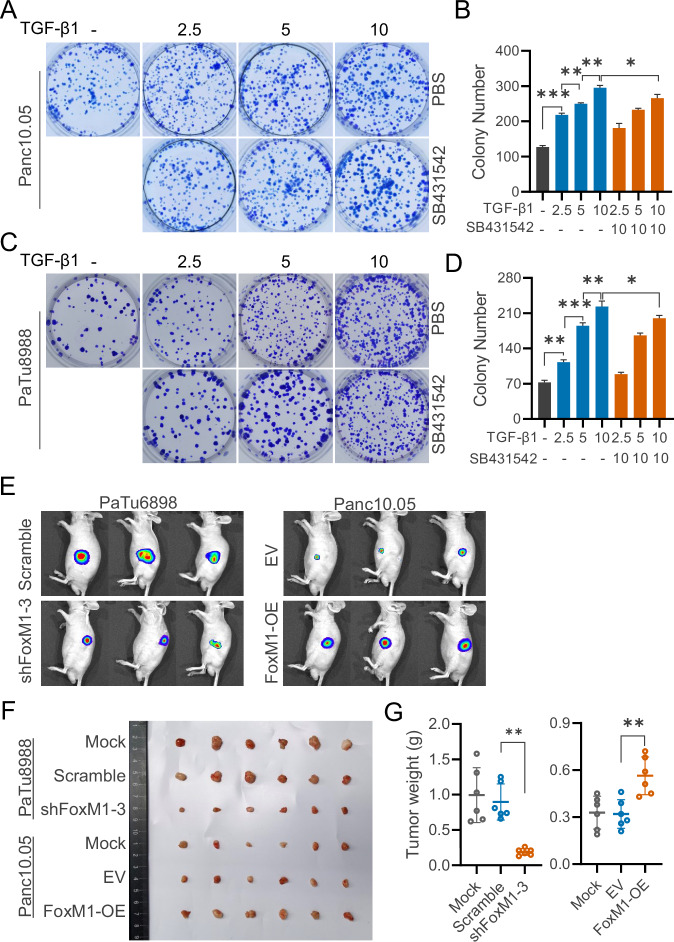
FoxM1 enhances TGF-β-driven pancreatic tumorigenesis in vitro and in vivo. Representative images and quantification of colony formation assay of Panc10.05 (**A**, **B**) and PaTu8988 (**C**, **D**) with or without TGF-β1 and SB431542 treatment. Representative images of luciferase activation in live mice with orthotopically-implanted pancreatic cancer cells at 5 weeks after transplantation (**E**). Representative images and tumor weights of pancreatic xenografts in BALB/c nude mice at day 49 post-orthotopic implantation (**F**, **G**). The data are presented as mean ± SD of triplicate quantification. * *p* < 0.05, ** *p* < 0.01, and *** *p* < 0.001.

To explore the role of FoxM1 in TGF-β1 signaling–dependent pancreatic tumorigenesis, we first examined the effect of TGF-β1 on colony formation by pancreatic cancer cells Panc10.05 (Fig. 2A, B) and PaTu8988 (Fig. 2C, D) in vitro. As TGF-β1 concentration increased, the number of colonies also increased, indicating a dose-dependent enhancement of tumorigenic potential. Co-treatment with the TGF-β receptor inhibitor SB-431542 partially reduced colony formation, suggesting that TGF-β1 promotes pancreatic cancer cell proliferation and tumorigenic behavior through the TGF-β/Smad signaling pathway.

To further validate the tumor-promoting role of FoxM1 in vivo, we examined its effect on orthotopic pancreatic tumorigenesis in nude mice. Pancreatic cancer cell lines with stable FoxM1 overexpression or knockdown were injected into the pancreas of immunodeficient nude mice, and tumor growth was monitored over a five-week period. In vivo imaging revealed that tumors formed by FoxM1 knockdown pancreatic cancer cells were significantly smaller compared to their respective control cells. In contrast, tumors formed by FoxM1-overexpressing pancreatic cancer cells were significantly larger (Fig. 2E). These findings were further supported by analysis of the harvested five-week tumors (Fig. 2F) and their corresponding weights (Fig. 2G). These results suggest that FoxM1 promotes pancreatic tumorigenesis in vivo, potentially through upregulation of the TGF-β/Smad signaling pathway.

### FoxM1 promotes Smad4 expression and nuclear localization in response to TGF-β signaling

**Fig. 3:**
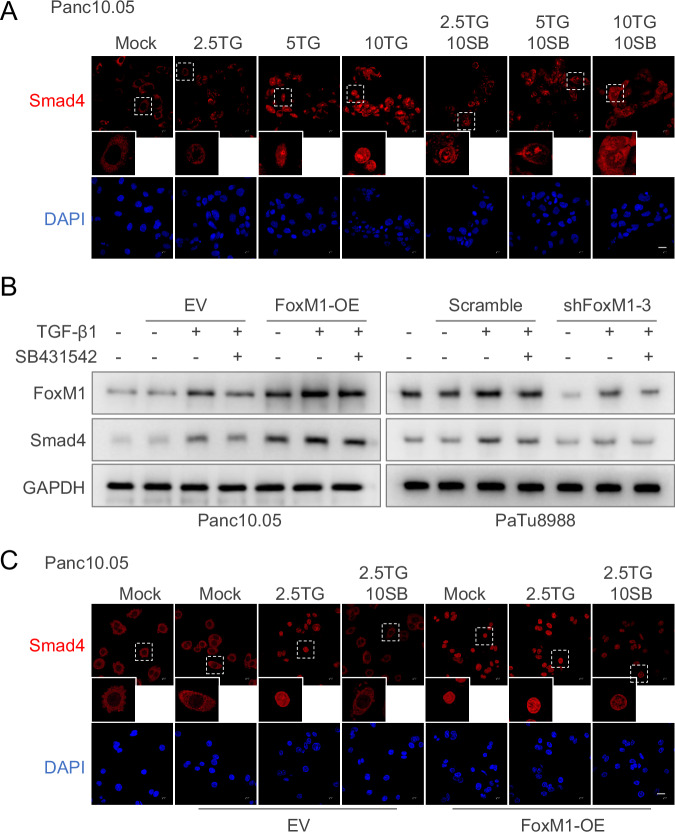
FoxM1 promotes Smad4 expression and nuclear localization in response to TGF-β signaling. **A** Representative immunostaining images of Panc10.05 cells treated with or without TGF-β1 and SB431542. **B** Western blot assay of FoxM1 and Smad4 expression in FoxM1-overexpressing Panc10.05 and FoxM1-knockdown Patu8999 cells treated with or without TGF-β1 and SB431542. **C** Immunofluorescence analysis of FoxM1-overexpressing Panc10.05 cells with or without TGF-β1 and SB431542 treatment. EV, empty vector. “2.5TG”, “5TG” and “10TG” represent for 2.5, 5 and 10 μg/ml TGF- β1, respectively. “10SB” means 10 μM SB431542. Scale bar: 20 μm.

To explore how TGF-β signaling modulates Smad4 in pancreatic cancer cells, we analyzed the expression levels and subcellular localization of Smad4 in Panc10.05 cells following TGF-β1 treatment (Fig. 3A). Immunofluorescence staining revealed that Smad4 translocated from the cytoplasm to the nucleus and nucleolus in a concentration-dependent manner upon TGF-β1 stimulation. This translocation was markedly inhibited by the TGF-β receptor inhibitor SB-431542. A similar pattern was observed in PaTu8988 cells (Fig. S4A).

To further investigate the role of FoxM1 in regulating Smad4 through the TGF-β pathway, we performed western blotting and immunofluorescence analyses using Panc10.05 cells overexpressing FoxM1 and PaTu8988 cells with FoxM1 knockdown. Western blot results showed that TGF-β1 upregulated Smad4 expression, an effect that was partially attenuated by SB-431542. Notably, FoxM1 overexpression further enhanced TGF-β1–induced Smad4 expression, whereas FoxM1 knockdown reduced it (Fig. 3B). Furthermore, FoxM1 overexpression alone was sufficient to promote Smad4 nuclear translocation, mimicking the effect of TGF-β1. Unlike TGF-β1–induced localization, this FoxM1-driven nuclear translocation was not reversed by SB-431542 (Figs. 3C and S4B).

To investigate whether FoxM1 knockdown affects additional signaling pathways involved in cell growth and cell cycle regulation, we analyzed the PI3K/AKT/mTOR pathway in FoxM1-deficient pancreatic cancer cells. Western blot analysis revealed that the phosphorylation of S6RP was increased, whereas pAKT levels remained largely unchanged (Fig. S4C). The increased S6RP phosphorylation indicates enhanced mTOR pathway activation upon FoxM1 silencing. However, this activation contrasts with the observed suppression of tumor cell proliferation, suggesting that FoxM1’s pro-tumorigenic function is not primarily mediated through growth-promoting pathways such as mTOR. Instead, our data support that FoxM1 exerts its oncogenic effects mainly by upregulating Smad4 expression and facilitating its nuclear translocation.

### FoxM1 enhances Smad4 stability and sustains TGF-β/Smad signaling

Building on our previous findings that FoxM1 promotes breast cancer metastasis by activating the TGF-β/Smad3 signaling pathway—through competition with Smad3 for TIF1γ binding and protection of Smad4 from ubiquitin-mediated degradation [11], we sought to determine whether a similar mechanism operates in pancreatic cancer cells.

**Fig. 4:**
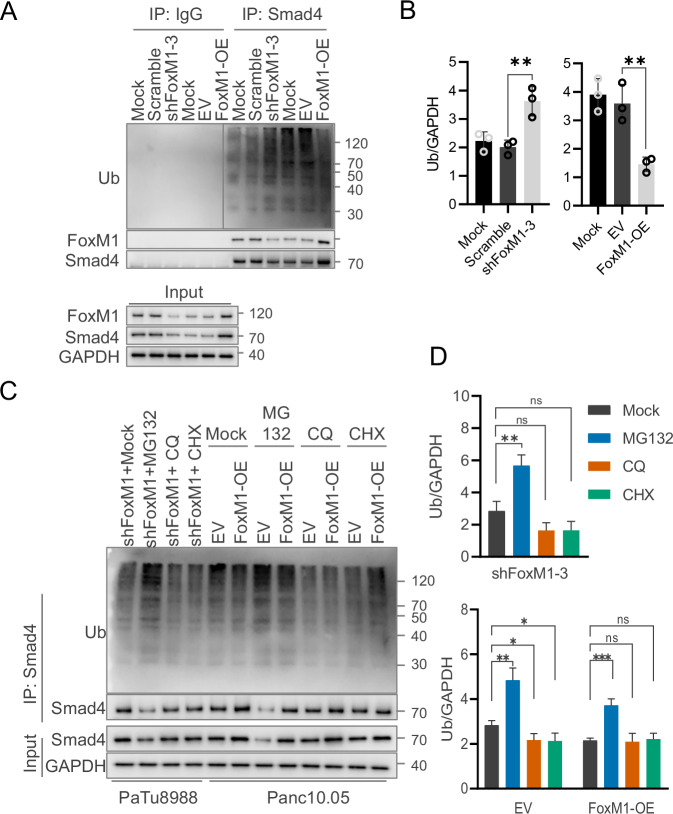
FoxM1 physically interacts with Smad4 and enhances its protein stability by inhibiting degradation. **A** Interaction of FoxM1 with ubiquitin in FoxM1 knockdown PaTu8988 and overexpressed Panc10.05 cells detected by Co-immunoprecipitation. **B** Quantification of ubiquitin pulled down, normalized by the corresponding input GAPDH. **C** Investigation of the interaction between FoxM1 and ubiquitin in Panc10.05 cells overexpressing FoxM1 and PaTu8988 cells with FoxM1 knockdown, with or without treatment using MG132, chloroquine (CQ), and cycloheximide (CHX). **D** Quantification of ubiquitin pulled down, normalized by the corresponding input GAPDH. The data are presented as mean ± SD of triplicate quantification. “ns” not significant, “*”*p* < 0.05, “**”*p* < 0.01, and “***”*p* < 0.001.

To investigate this, we performed co-immunoprecipitation assays in PaTu8988 and Panc10.05 cells with stable knockdown and overexpression of FoxM1 to assess the interaction between FoxM1 and Smad4. The results demonstrated that Smad4 physically interacts with FoxM1, and this interaction was strengthened by FoxM1 overexpression and weakened by FoxM1 knockdown. Correspondingly, levels of ubiquitinated Smad4 were inversely correlated with FoxM1 expression, suggesting that FoxM1 overexpression reduces Smad4 ubiquitination and thereby inhibits its degradation (Fig. 4A, B).

To further clarify the degradation pathway involved, we treated the cells with the proteasome inhibitor MG132, the lysosomal inhibitor chloroquine (CQ), and the protein synthesis inhibitor cycloheximide (CHX). MG132 treatment led to an accumulation of polyubiquitinated Smad4 (Fig. 4C, D), supporting the conclusion that Smad4 degradation occurs primarily via the proteasome. Together, these findings indicate that FoxM1 stabilizes Smad4 by protecting it from proteasomal degradation, thereby enhancing TGF-β/Smad pathway activity in pancreatic cancer cells.

### FoxM1 and Smad4 reciprocally regulate transcription via direct promoter binding

**Fig. 5:**
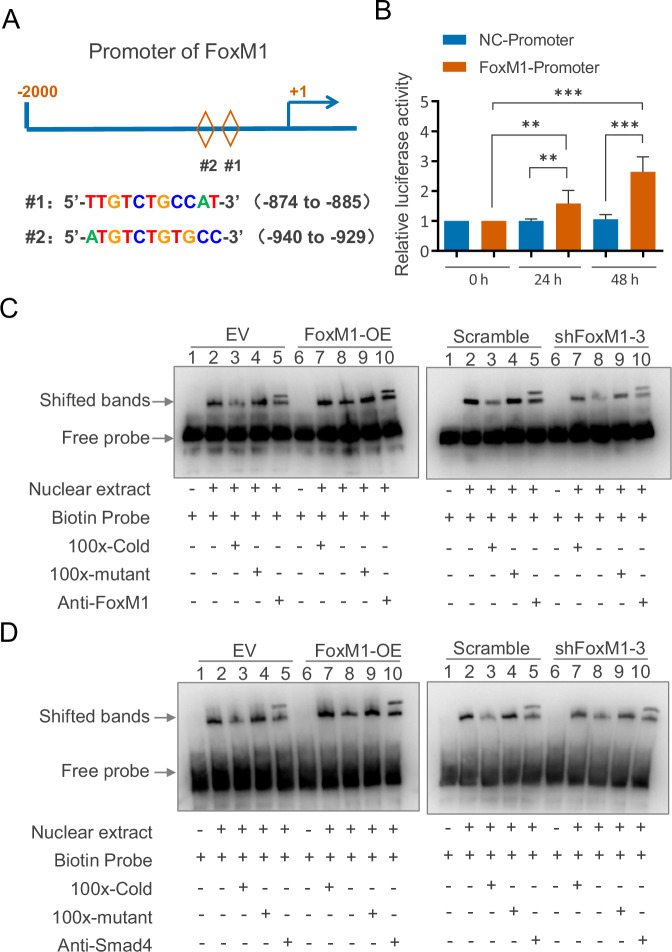
Reciprocal promoter binding between FoxM1 and Smad4. **A** The predicted two binding sites of SMAD4 on the FoxM1 promoter area. **B** Luciferase activity analysis of human FoxM1 promoter. BxPC3 cells were transfected with GV238 Renilla-Luciferase-expressing vector containing the regions of the 5’-flanking sequence of the FoxM1 gene and co-transfected with SMAD4. After 0 h, 24 h and 48 h, the cells were lysed and assayed for luciferase activity. The luciferase activity of each individual transfectant was subsequently normalized to Renilla luciferase activity. The results were expressed as the relative firefly luciferase activity ratio to Renilla luciferase activity. Data are the mean ± SD (%) of three independent analyses. Electrophoretic mobility shift assay (EMSA) was performed to test the interaction of FoxM1 with Smad4’s promoter probes (**C**) and the interaction of Smad4 with FoxM1’s promoter probes (**D**).

To determine whether FoxM1 regulates the transcription of Smad4, we conducted a bioinformatics analysis and identified two potential Smad4-binding sites within the FoxM1 promoter region, located at positions –874 to –885 bp and –940 to –929 bp upstream of the transcription start site (Fig. 5A). Consistently, analysis of ENCODE ChIP-seq datasets from K562, WTC11 and HepG2 cell lines with CRISPR-mediated SMAD4 insertion revealed clear SMAD4 enrichment peaks at the FoxM1 promoter region (Fig. S5). Furthermore, luciferase reporter assays showed that co-transfection of a Smad4 overexpression plasmid with a FoxM1 promoter-luciferase construct significantly enhanced FoxM1 promoter activity (Fig. 5B), suggesting that Smad4 can transcriptionally activate FoxM1.

To investigate whether FoxM1 and Smad4 directly bind to each other’s promoter regions, we performed electrophoretic mobility shift assays (EMSA). The results demonstrated that FoxM1 protein binds to the Smad4 promoter, as shown by a reduction in signal upon competition with a non-biotinylated wild-type probe, but not with a mutant probe. Binding activity increased with FoxM1 overexpression and decreased with FoxM1 knockdown. Addition of a FoxM1 antibody caused a supershift, further confirming specific interaction (Fig. 5C). Similarly, EMSA analysis revealed that Smad4 can bind directly to the FoxM1 promoter (Fig. 5D). These results support a model in which FoxM1 and Smad4 reciprocally bind to and activate each other’s promoters, forming a positive transcriptional feedback loop.

### Existence of FoxM1-Smad4 positive feedback loop in pancreatic tumor tissues

**Fig. 6:**
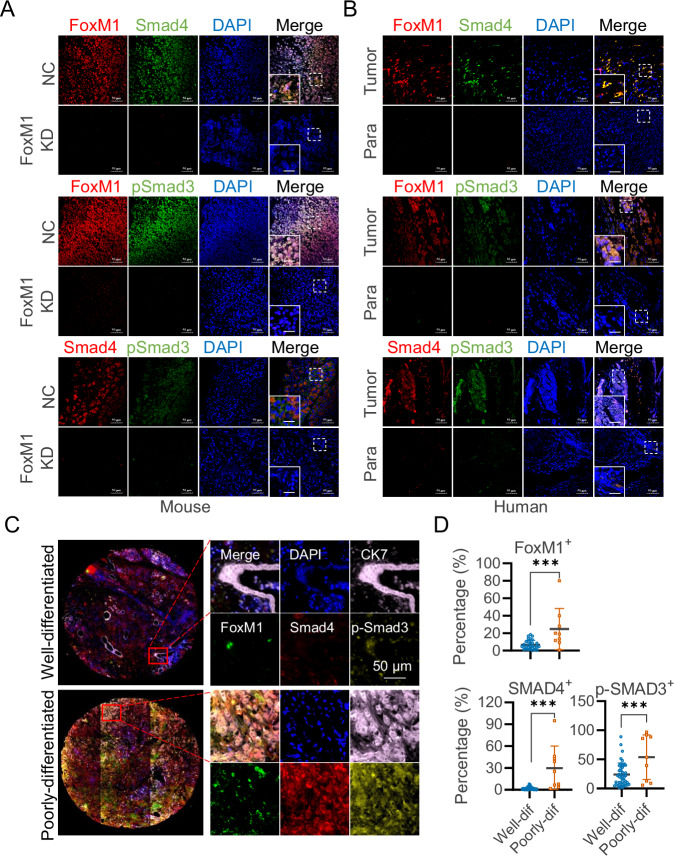
Co-localization and expression of Smad4 and pSmad3 in FoxM1 knockdown pancreatic xenografts and human pancreatic cancer tissues. **A** The expression of Smad4 and phosphorylated Smad3 is markedly reduced in FoxM1 knockdown PaTu8988 tumor xenografts. Scale bar: 50 μm. **B** Representative immunostaining image of human pancreatic tissue, showing a comparison between tumor and adjacent non-cancerous (paracancerous) tissues. Scale bar: 50 μm. The analysis reveals a significant reduction in the levels of Smad4 and phosphorylated Smad3 in the paracancerous tissues. **C** Representative multiplex immunostaining images of well-differentiated and poorly-differentiated pancreatic cancer tissues. Scale bar: 50 μm. **D** Quantification of the percentage of FoxM1^+^, SMAD4^+^, and p-SMAD3^+^ positive cells relative to CK7^+^ tumor cells. Each data point represents an individual patient, with percentages calculated as the average from three randomly selected fields containing over 100 tumor cells each. *** *p* < 0.001.

To confirm the presence of a FoxM1-Smad4 positive feedback loop in vivo, we analyzed tumor tissues derived from FoxM1-knockdown Patu8988 xenografts and their corresponding control tumors. Immunofluorescence was used to assess the expression levels of FoxM1, Smad4, and p-Smad3. Tumors with FoxM1 knockdown exhibited markedly reduced expression of all three proteins, supporting a regulatory relationship among them (Fig. 6A).

To evaluate the clinical relevance of this feedback loop, we next examined the expression of FoxM1, Smad4, and p-Smad3 in tumor tissues from pancreatic cancer patients. Expression levels of these proteins were consistently lower in adjacent non-tumor (paratumoral) tissues compared to matched tumor samples (Fig. 6B). To expand our analysis, we performed multiplex immunofluorescence staining on human pancreatic cancer tissue microarrays, using CK7 co-staining to delineate tumor regions (Fig. 6C). Notably, FoxM1, Smad4, and p-Smad3 levels were significantly elevated in poorly differentiated tumors compared to well-differentiated ones (Fig. 6D and Tables S1–S3). These findings provide in vivo and clinical evidence for the existence of a FoxM1–Smad4 positive feedback loop, suggesting its functional role in pancreatic tumor progression.

**Fig. 7:**
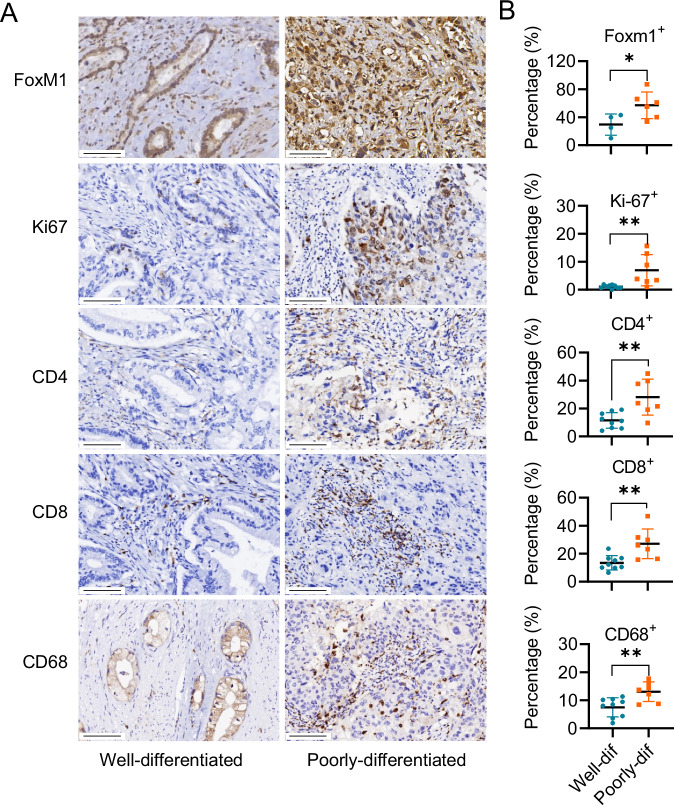
FoxM1 is positively associated with immune cell infiltration and tumoral proliferation. **A** Representative IHC staining of FoxM1, Ki67, CD4, CD8 and CD68 on well- and poorly-differentiated tumor tissues of human pancreatic cancer patients. **B** Quantification of the percentage of FoxM1^+^, Ki67^+^, CD4^+^, CD8^+^ and CD68^+^ cells relative to total cells. Each data point represents an individual patient, with percentages calculated as the average from three randomly selected fields. * *p* < 0.05 and ** *p* < 0.01.

To address the potential influence of immune interactions and microenvironmental factors on human pancreatic tumor tissues, we analyzed clinical pancreatic cancer specimens with varying differentiation grades for the expression of FoxM1, Ki67, and immune cell markers, including CD4, CD8, and CD68. Compared with well-differentiated tumors, poorly differentiated pancreatic cancers exhibited higher FoxM1 and Ki67 expression, accompanied by increased infiltration of T cells, B cells, and macrophages, suggesting enhanced immune activity within these tumor tissues (Fig. 7).

In summary, FoxM1 regulates Smad4 through two complementary mechanisms: it enhances Smad4 transcription by binding to its promoter region and stabilizes Smad4 protein via direct interaction, preventing its degradation. Conversely, Smad4 also binds to the FoxM1 promoter, promoting FoxM1 transcription. This reciprocal regulation establishes a positive feedback loop between FoxM1 and Smad4, which contributes to the sustained activation of TGF-β/Smad signaling and promotes pancreatic tumor progression.

## Discussion

Pancreatic cancer remains one of the most lethal malignancies worldwide, underscoring the urgent need to better understand its pathogenesis and identify effective therapeutic targets [1, 2]. In this study, we uncovered a FoxM1–Smad4 positive feedback signaling loop through a combination of molecular cytology techniques and pancreatic orthotopic transplantation models in mice. Mechanistically, FoxM1 stabilizes Smad4 by inhibiting its ubiquitin-dependent proteasomal degradation. In addition to functioning as a downstream effector of TGF-β signaling, FoxM1 also binds to the Smad4 promoter, enhancing its transcription. This reciprocal regulation forms a feedforward loop that amplifies TGF-β–mediated signaling, thereby promoting pancreatic tumorigenesis.

FoxM1, a key oncogenic transcription factor, has been shown to form positive feedback loops with various signaling molecules to drive cancer progression [15–19]. For example, recent studies revealed that the tyrosine kinase c-Src activates FoxM1, establishing a feedback loop that promotes proliferation in both genetically engineered and patient-derived models of Luminal B-like breast cancer [19]. Similarly, in acute myeloid leukemia (AML), FoxM1 and AKT form an autoregulatory loop that maintains high activity of both pathways; disruption of either component leads to coordinated downregulation of the other and breakdown of the loop [20]. Moreover, overexpression of Dickkopf-1 (DKK1) enhances tumor growth in pancreatic and esophageal cancers by binding to cytoskeleton-associated protein 4 (CKAP4) [21]. In this context, our study identifies a novel FoxM1–Smad4 positive feedback loop that promotes pancreatic tumorigenesis, representing a new dimension of FoxM1’s oncogenic role and functional versatility.

Despite the valuable insights gained, this study has certain limitations. While we demonstrate the tumor-promoting role of the FoxM1–Smad4 positive feedback loop in pancreatic cancer, it is important to acknowledge that FoxM1 may also contribute to tumorigenesis and metastasis through alternative signaling pathways or regulatory circuits, such as the AKT pathway [20], β-catenin [22], or p65 signaling [23]. Furthermore, Smad family proteins themselves are highly dynamic and capable of forming additional feedback loops that facilitate tumor progression [24–26]. For example, Smads have been shown to interact with lnc-UTGF to enhance liver cancer metastasis [27] and with MYOCD to drive TGF-β-induced epithelial-mesenchymal transition in non-small cell lung cancer [24]. It is also worth noting that several of the cell lines used in this study harbor TP53 mutations or deletions. For instance, the FoxM1-high Panc-1 line carries the DNA-contact mutant R273H, which disrupts sequence-specific DNA binding and exhibits strong dominant-negative and gain-of-function activities. PaTu8988-T contains the hotspot mutation R282W, a structural/conformational defect that destabilizes the DNA-binding domain through a distinct mechanism [28, 29]. In contrast, the FoxM1-low AsPC-1 line is TP53-null [30]. Although TP53 status was not a primary criterion in our cell-line selection, the use of models with different TP53 mutation backgrounds represents a limitation and may introduce complexity into the interpretation of FoxM1–SMAD4–mediated phenotypes. Whether the subtle functional differences between R282W and R273H influence the FoxM1–Smad regulatory circuit in pancreatic cancer remains an open question and warrants further investigation. Future studies incorporating isogenic TP53-wildtype and TP53-mutant systems, or TP53 restoration models, will be valuable for clarifying whether TP53 directly influences the hierarchy or directionality of the FoxM1–SMAD4 positive feedback loop. Therefore, these limitations underscore the broader complexity of the regulatory networks involved and highlight the need for further investigation into additional co-regulatory mechanisms involving FoxM1 and Smad proteins.

## Supplementary information

Supplemental Figures

Supplementary Figure Legends

Tables S1 S2 S3

Original Data

5-checklist

## Data Availability

The ChIP-seq datasets analyzed during the current study are available in the ENCODE repository (https://www.encodeproject.org/). All other data generated or analyzed during this study are included in this published article and its supplementary information files. Requests for resources, raw data, or further information should be directed to and will be fulfilled by the lead contacts, Shaojiang Zheng (zshaojiang@muhn.edu.cn) and Yanda Lu (luyandajx@sina.com). This study did not generate new, unique reagents.
