# Supplementary figures and images for: A FoxM1/Smad4 positive feedback loop promotes pancreatic cancer progression
Source: Cell Death Dis. 2026 Apr 10;17(1):465. doi: 10.1038/s41419-026-08697-y (PMC13181101; doi:10.1038/s41419-026-08697-y)

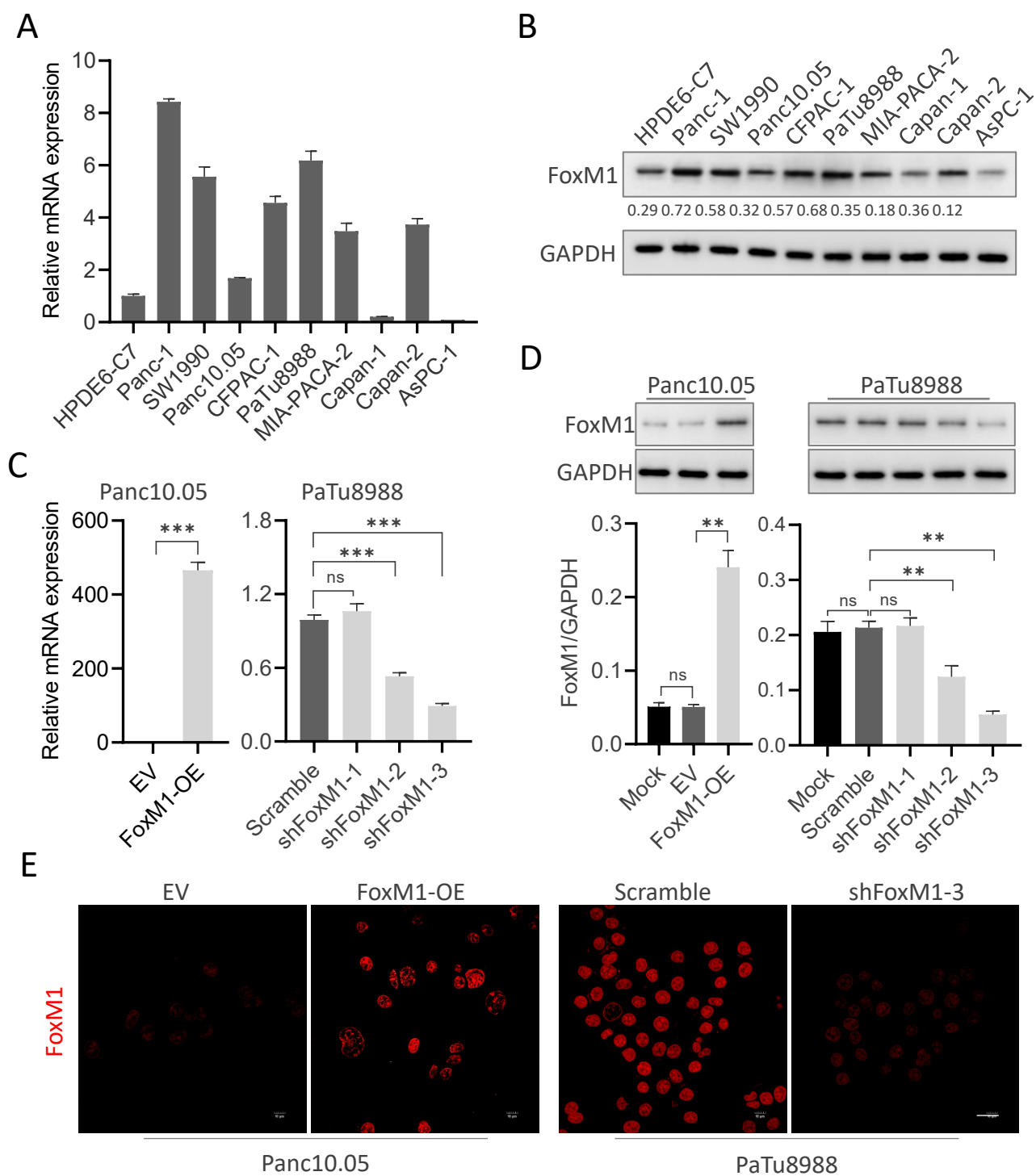

Fig S1

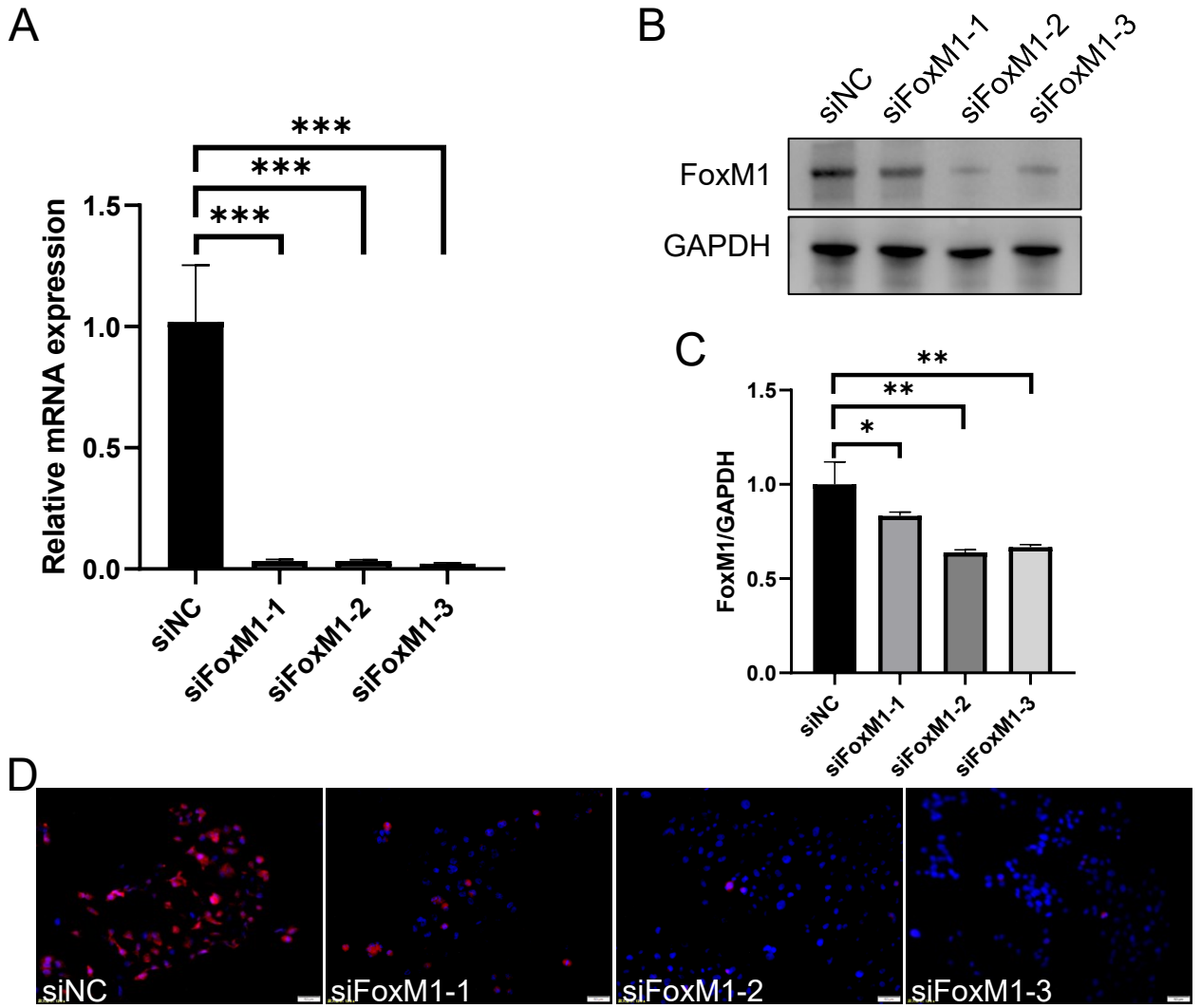

Fig S2

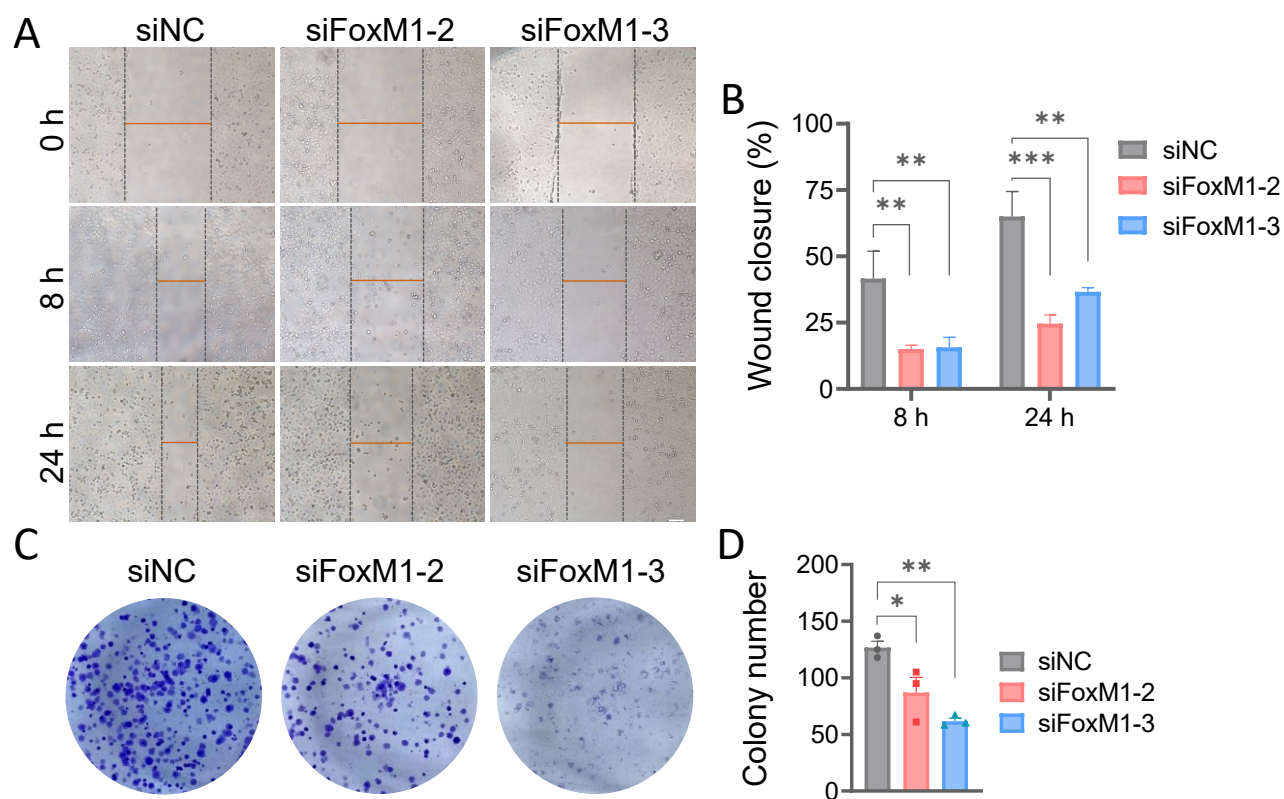

Fig S3

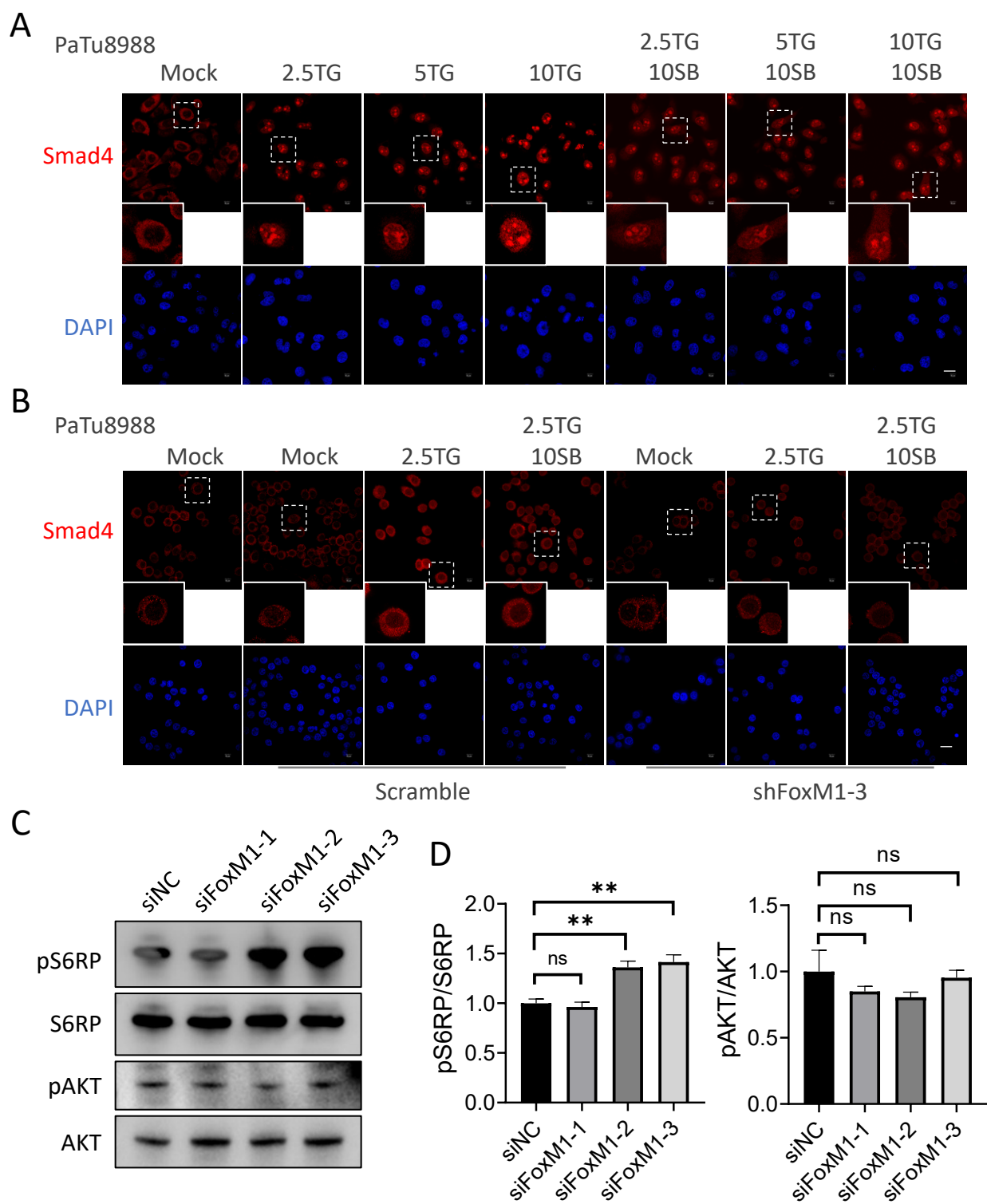

Fig S4

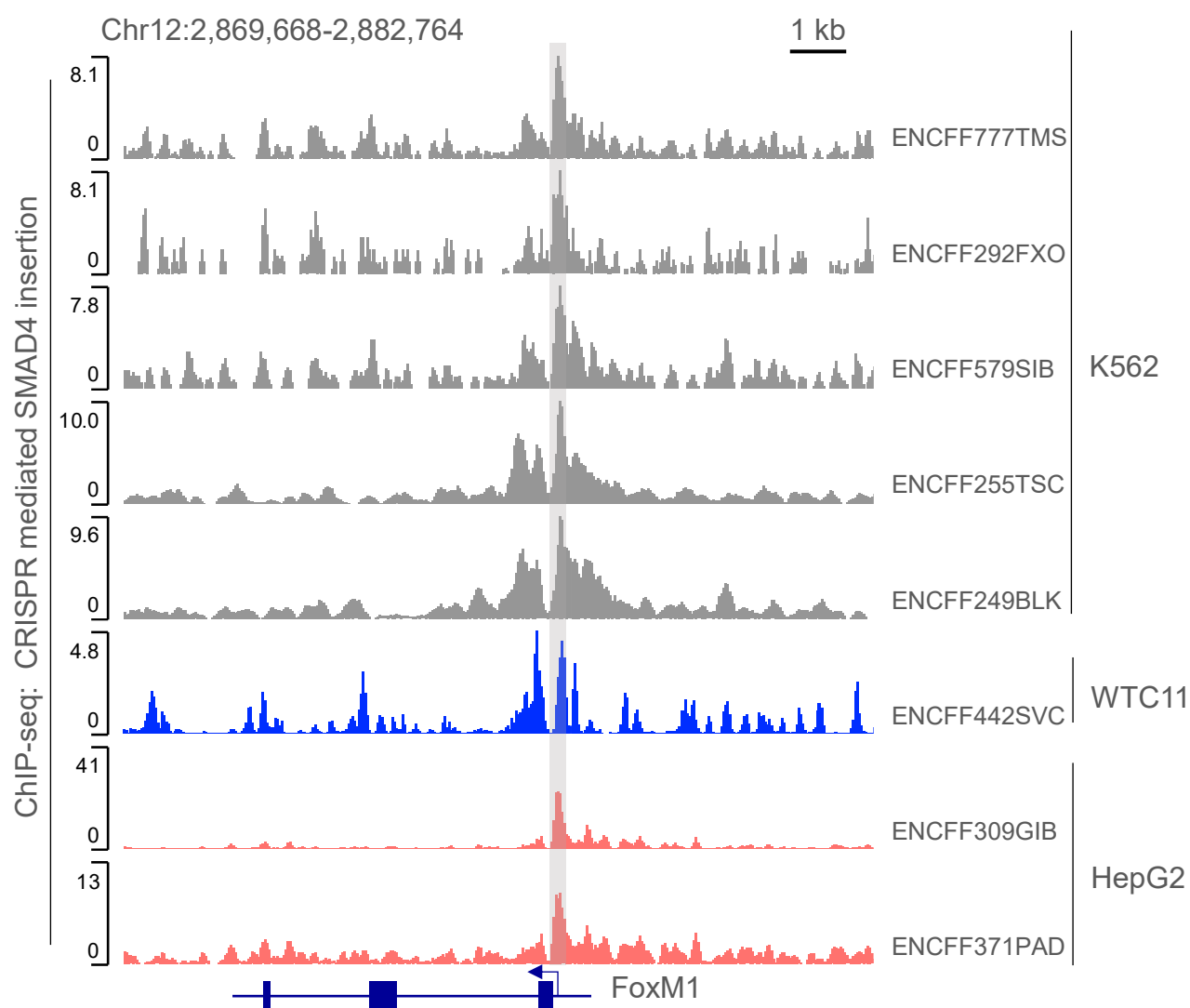

Fig S5

Supplement: Supplementary file 1 — Supplemental Figures [file 41419_2026_8697_MOESM1_ESM.pdf]
