# Supplementary material for: A FoxM1/Smad4 positive feedback loop promotes pancreatic cancer progression
Source: Cell Death Dis. 2026 Apr 10;17(1):465. doi: 10.1038/s41419-026-08697-y (PMC13181101; doi:10.1038/s41419-026-08697-y)
Supplement: Supplementary file 2 — Supplementary Figure Legends [file 41419_2026_8697_MOESM2_ESM.docx]

**Figure S1** **Modulation of FoxM1 expression by knockdown and overexpression in pancreatic cancer cells.** The expression of FoxM1 was measured in 10 different pancreatic tumor cell lines using Real-time qPCR (**A**) and Western blotting assay (**B**). The high FoxM1 cell line PaTu8988 and the low FoxM1 cell line Panc10.05 were used for FoxM1 shRNA knockdown and overexpression, respectively. FoxM1 expression was confirmed by Real-time qPCR (**C**), Western blotting (**D**), and Immunofluorescent staining (**E**). Data are presented as mean ± SD of triplicate quantification. ns, not significant, ** *p* < 0.01, and *** *p* < 0.001. Scale bar = 10 μm.

**Figure S2 FoxM1 knockdown by siRNAs promoted mTOR pathway activation in Patu8988 pancreatic cancer cells.** Three independent siRNAs targeting FoxM1 were used. FoxM1 expression was validated by Real-time qPCR **(A)**, Western blotting (**B and C**) and Immunofluorescent staining (**D**). The relative expression of FoxM1 to the internal control GAPDH was quantified to confirm knockdown efficiency. The data are presented as mean ± SD from three independent experiments. * *p* < 0.05, ** *p* < 0.01, and *** *p* < 0.001.

**Figure S3 FoxM1 depletion inhibited cell migration and colony formation.** A **s**cratch assay was performed to evaluate cell migration. Representative images of the scratch experiment (**A**) and colony formation assay (**C**) are shown, along with quantification of wound closure percentage (**B**) and colony number (**D**). The data are presented as mean ± SD of triplicate quantification. * *p* < 0.05, ** *p* < 0.01, and *** *p* < 0.001.

**Figure S4** **FoxM1 promotes TGF-β-mediated Smad4 expression and nucleus localization.** (**A**) Representative immunostaining images of PaTu8988 cells treated with or without TGF-β1 and SB431542. (**B**) Immunofluorescence analysis of FoxM1-knockdown PaTu8988 cells with or without TGF-β1 and SB431542 treatment. EV, empty vector. “2.5TG”, “5TG” and “10TG” represent for 2.5, 5 and 10 μg/ml TGF- β1, respectively. “10SB” means 10 μM SB431542. Scale bar: 20 μm. (**C**) Immunoblotting analysis of PI3K/AKT/mTOR pathway. Phosphorylation of S6RP relative to total S6RP was used as an indicator of mTOR pathway activation, while phosphorylation of AKT relative to total AKT reflected PI3K/AKT pathway activity. The data are presented as mean ± SD from three independent experiments. ns, not significant, * *p* < 0.05, ** *p* < 0.01, and *** *p* < 0.001.

**Figure S5 Genome browser visualization of SMAD4 ChIP–seq profiles in CRISPR-mediated SMAD4 insertion cell lines.** ChIP–seq tracks from ENCODE datasets (<https://www.encodeproject.org/>) show SMAD4 enrichment at the promoter area of FOXM1 in K562, WTC11, and HepG2 human cell lines following CRISPR-mediated SMAD4 insertion. Gray shading indicates promoter-like areas with SMAD4 binding.
