# Supplementary material for: A FoxM1/Smad4 positive feedback loop promotes pancreatic cancer progression
Source: Cell Death Dis. 2026 Apr 10;17(1):465. doi: 10.1038/s41419-026-08697-y (PMC13181101; doi:10.1038/s41419-026-08697-y)
Supplement: Supplementary file 3 — Tables S1 S2 S3 [file 41419_2026_8697_MOESM3_ESM.docx]

**Table S1. Relationship between FoxM1 protein expression and clinical pathological parameters of PDAC**

| **Clinical parameters** |  | **n** | **FoxM1 level（average）** | ***p*-value** |
| --- | --- | --- | --- | --- |
| Age | ＜60 | 33 | 10.87 | 0.0168 |
|  | ≥60 | 31 | 24.74 |  |
| Gender | Male | 34 | 15.02 | 0.3557 |
|  | Female | 30 | 20.5 |  |
| Differentiation | High+Moderate | 49 | 11.75 | 0.0002 |
|  | Low | 15 | 36.66 |  |
| TNM Stage | I + II | 35 | 16.11 | 0.5832 |
|  | III + IV | 29 | 19.37 |  |
| Vascular invasion | No | 39 | 13.69 | 0.0966 |
|  | Yes | 26 | 23.68 |  |
| Nerve invasion | No | 26 | 15.46 | 0.4664 |
|  | Yes | 36 | 19.94 |  |

**Table S2. Relationship between Smad3 protein expression and clinical pathological parameters of PDAC**

| **Clinical parameters** |  | **n** | **Smad3 level（average）** | ***p*-value** |
| --- | --- | --- | --- | --- |
| Age | ＜60 | 32 | 33.43 | 0.0283 |
|  | ≥60 | 29 | 50.58 |  |
| Gender | Male | 32 | 40.06 | 0.6869 |
|  | Female | 29 | 43.27 |  |
| Differentiation | High+Moderate | 49 | 35.87 | 0.0122 |
|  | Low | 12 | 61.25 |  |
| TNM Stage | I + II | 33 | 34.87 | 0.0635 |
|  | III + IV | 28 | 49.05 |  |
| Vascular invasion | No | 39 | 37.81 | 0.1649 |
|  | Yes | 21 | 49.52 |  |
| Nerve invasion | No | 27 | 40.77 | 0.7927 |
|  | Yes | 33 | 42.90 |  |

**Table S3. Relationship between Smad4 protein expression and clinical pathological parameters of PDAC**

| **Clinical parameters** |  | **n** | **Smad4 level（average）** | ***p*-value** |
| --- | --- | --- | --- | --- |
| Age | ＜60 | 33 | 13.03 | 0.2568 |
|  | ≥60 | 31 | 20.58 |  |
| Gender | Male | 34 | 18.38 | 0.5892 |
|  | Female | 30 | 14.76 |  |
| Differentiation | High+Moderate | 49 | 10.34 | 0.0003 |
|  | Low | 15 | 37.4 |  |
| TNM Stage | I + II | 35 | 15.68 | 0.7420 |
|  | III + IV | 29 | 18.5 |  |
| Vascular invasion | No | 40 | 15.95 | 0.6251 |
|  | Yes | 22 | 19.45 |  |
| Nerve invasion | No | 26 | 15.95 | 0.5035 |
|  | Yes | 37 | 18.65 |  |
