# Supplementary material for: A FoxM1/Smad4 positive feedback loop promotes pancreatic cancer progression
Source: Cell Death Dis. 2026 Apr 10;17(1):465. doi: 10.1038/s41419-026-08697-y (PMC13181101; doi:10.1038/s41419-026-08697-y)
Supplement: Supplementary file 4 — Original Data [file 41419_2026_8697_MOESM4_ESM.pdf]

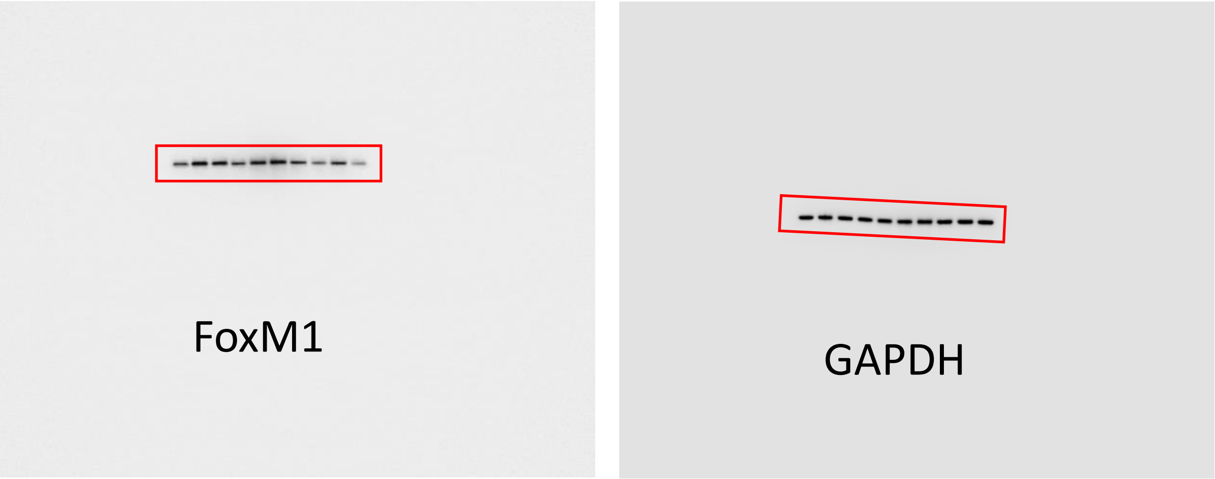

Figure 1B

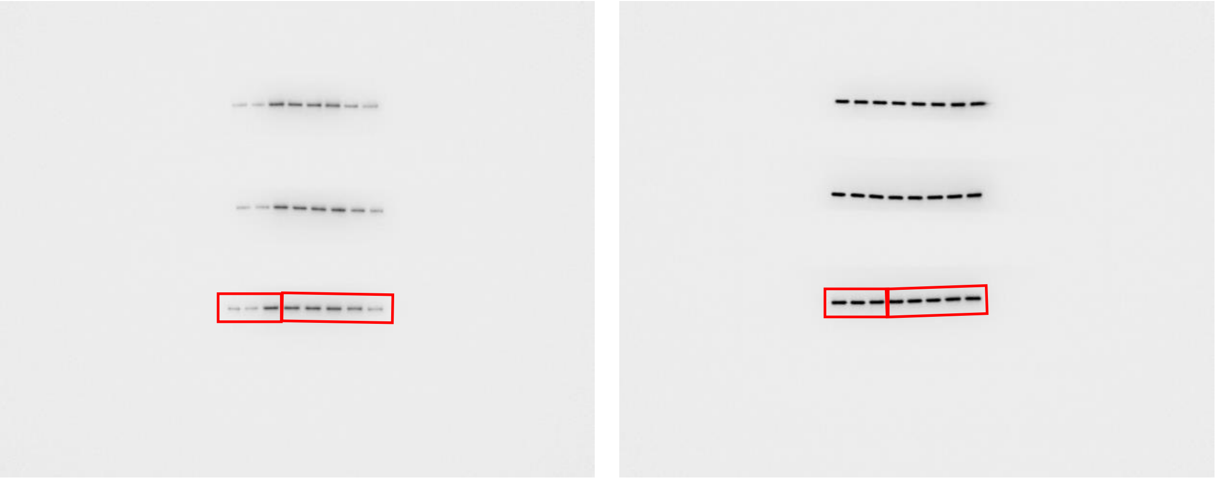
Figure 1D

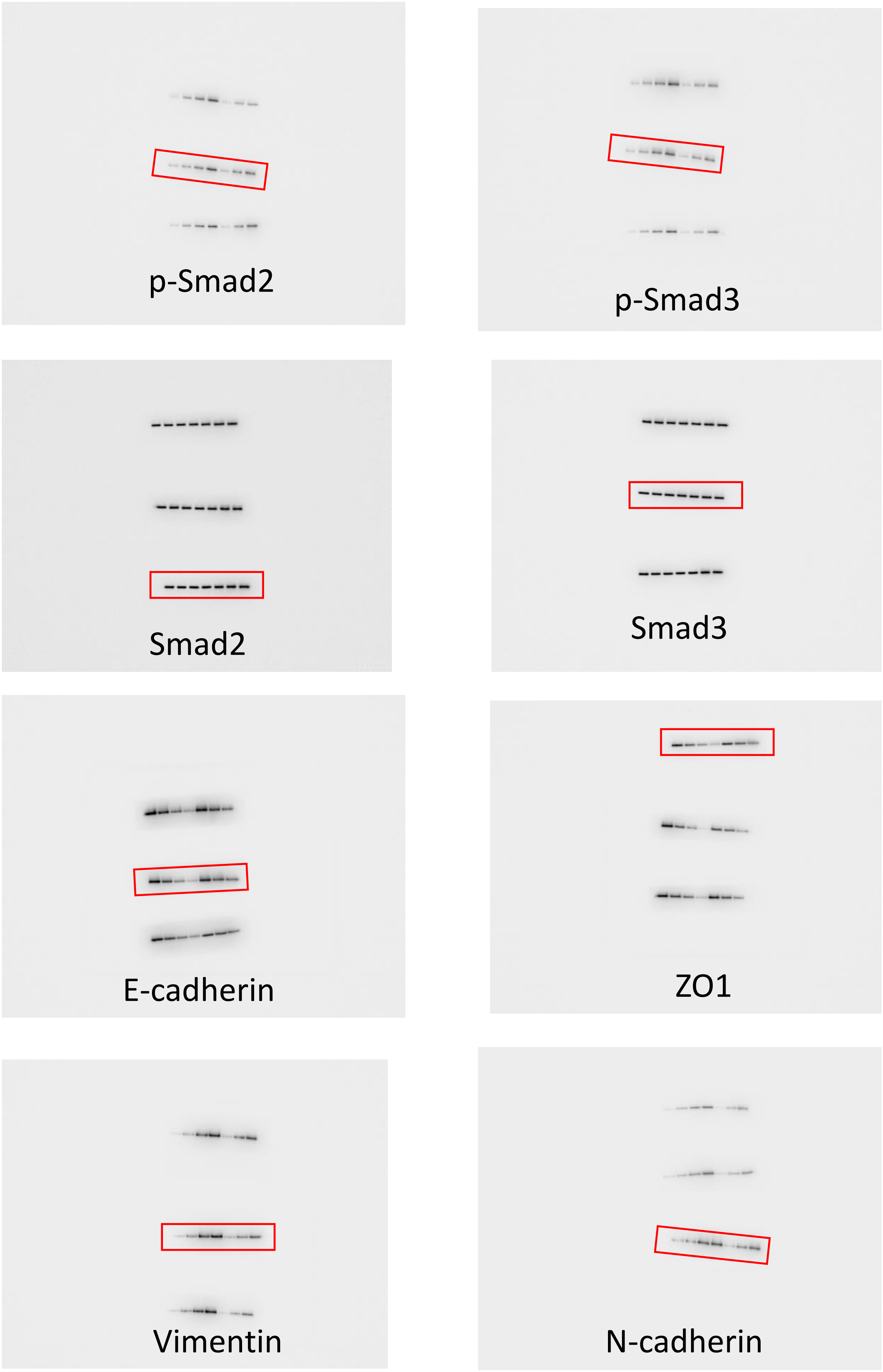
p-Smad2
p-Smad3
Smad2
Smad3
E-cadherin
ZO1
Vimentin
N-cadherin

Figure 2A

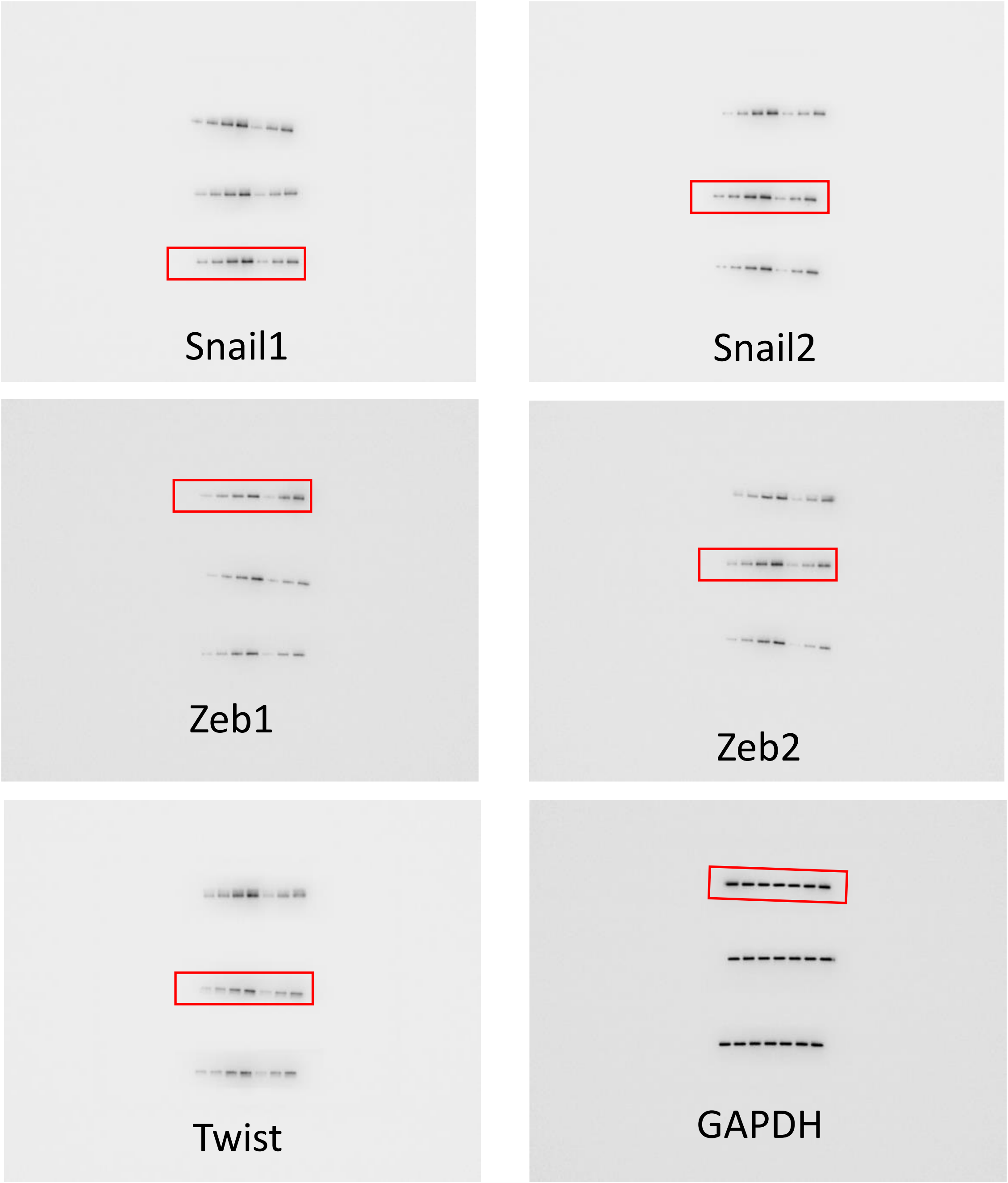

Figure 2A

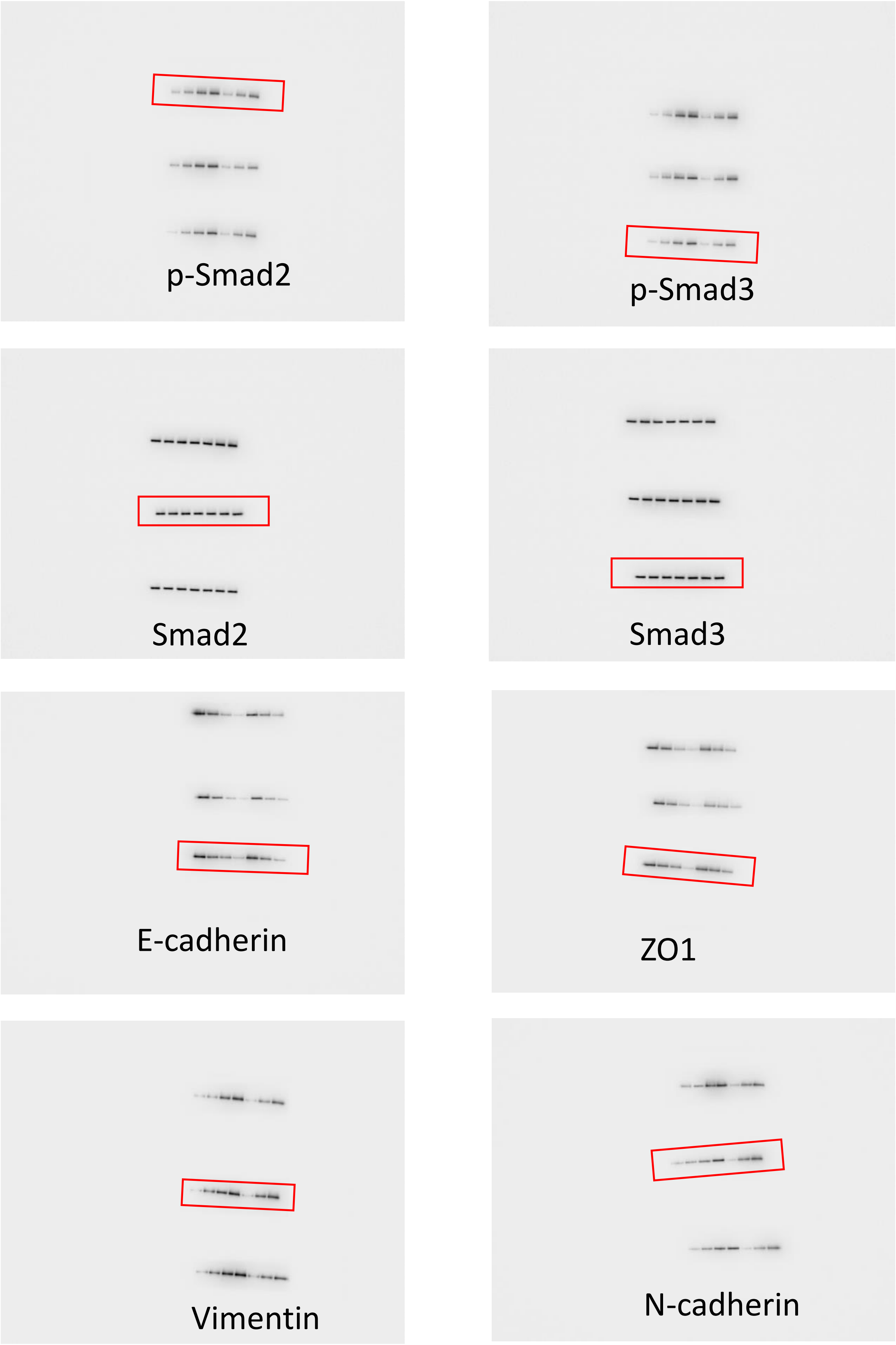
p-Smad2
p-Smad3
Smad2
Smad3
E-cadherin
ZO1
Vimentin
N-cadherin

Figure 2B

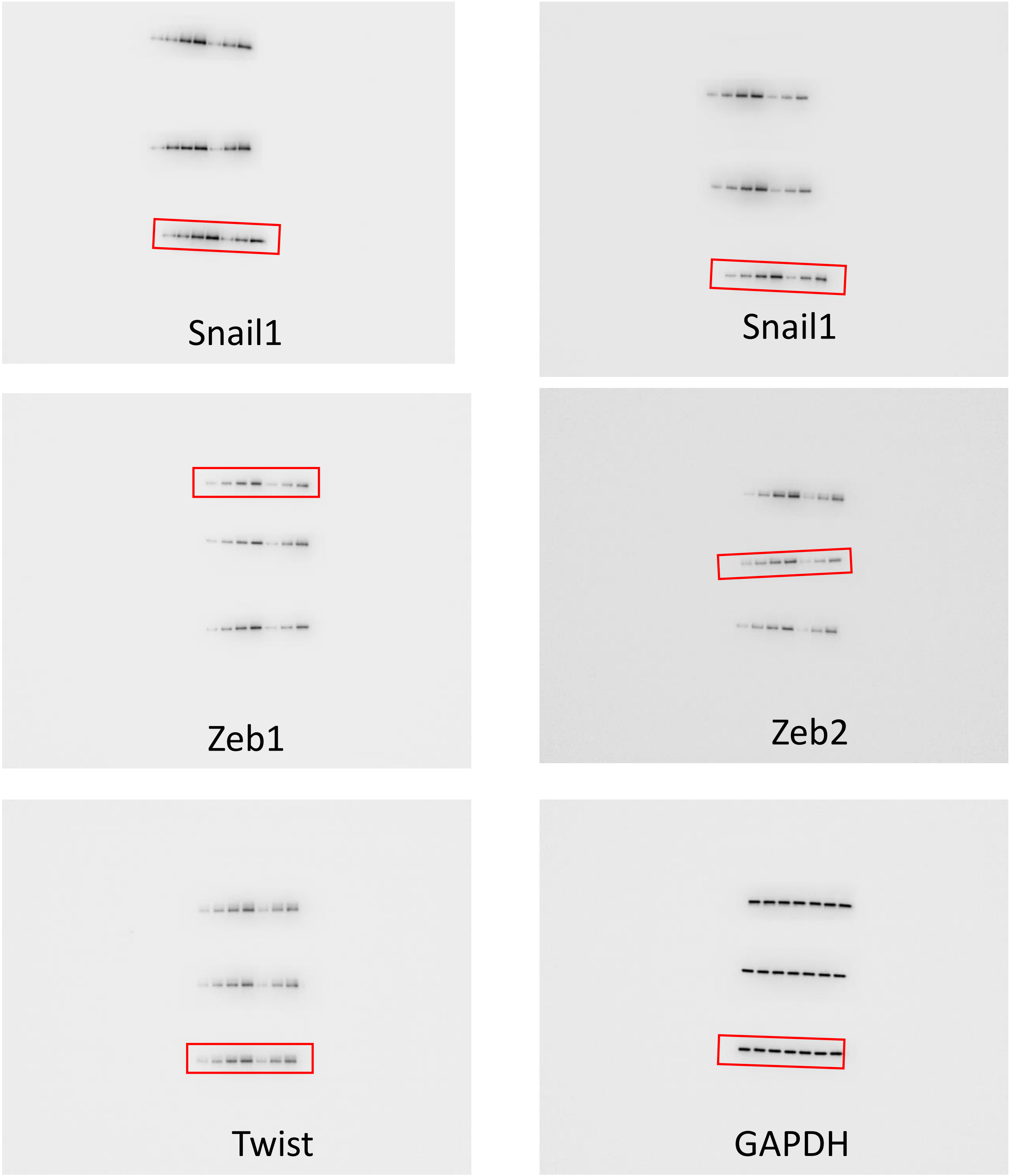

Figure 2B

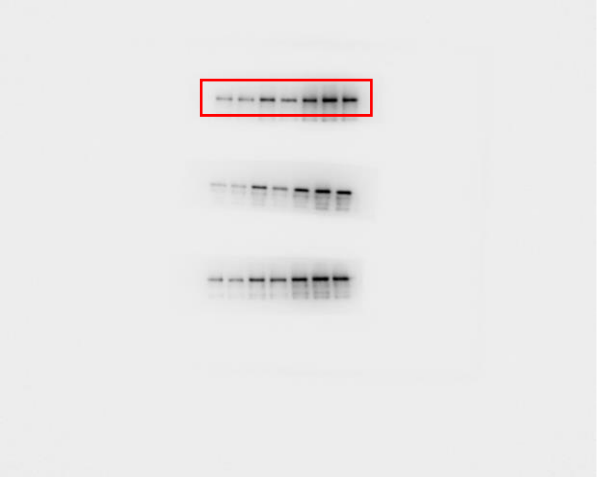

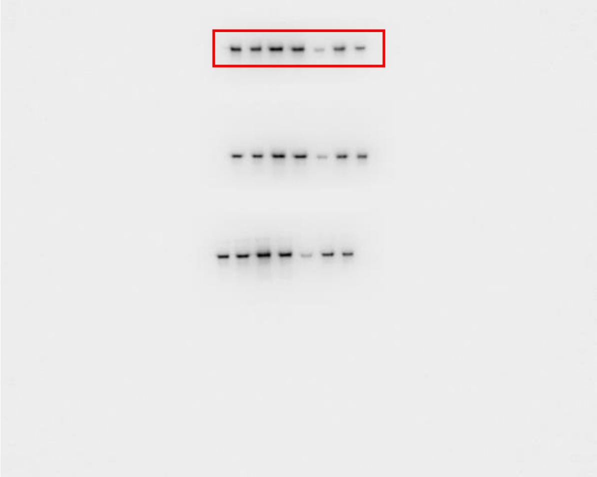

FoxM1 FoxM1

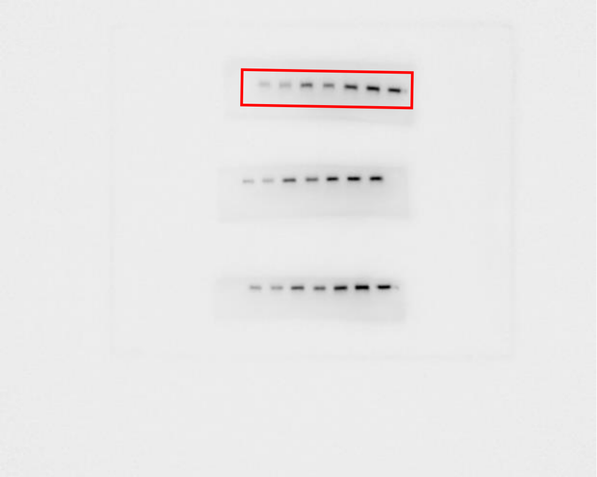

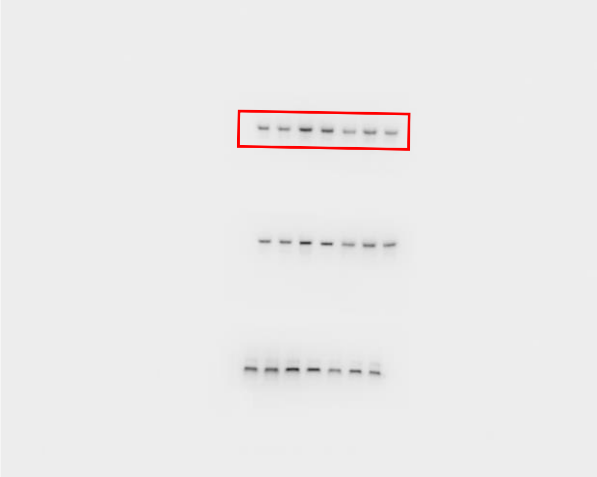

Smad4 Smad4

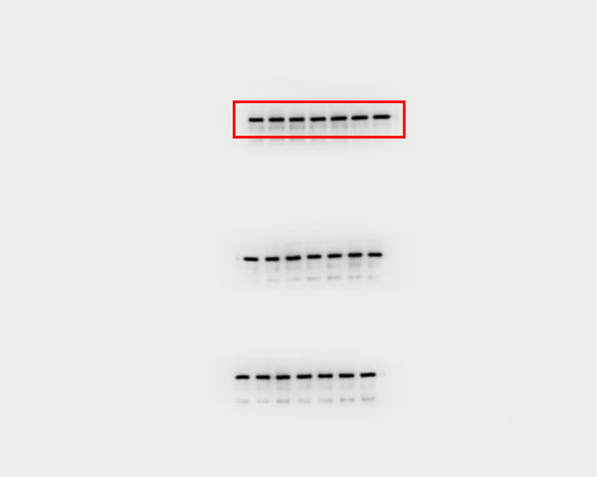

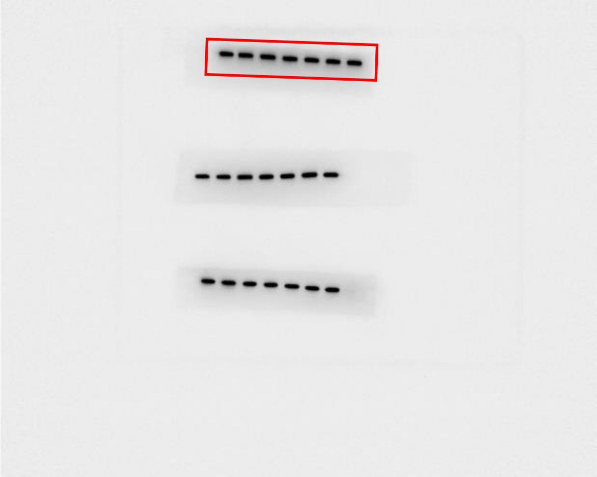

GAPDH GAPDH

Figure 4B

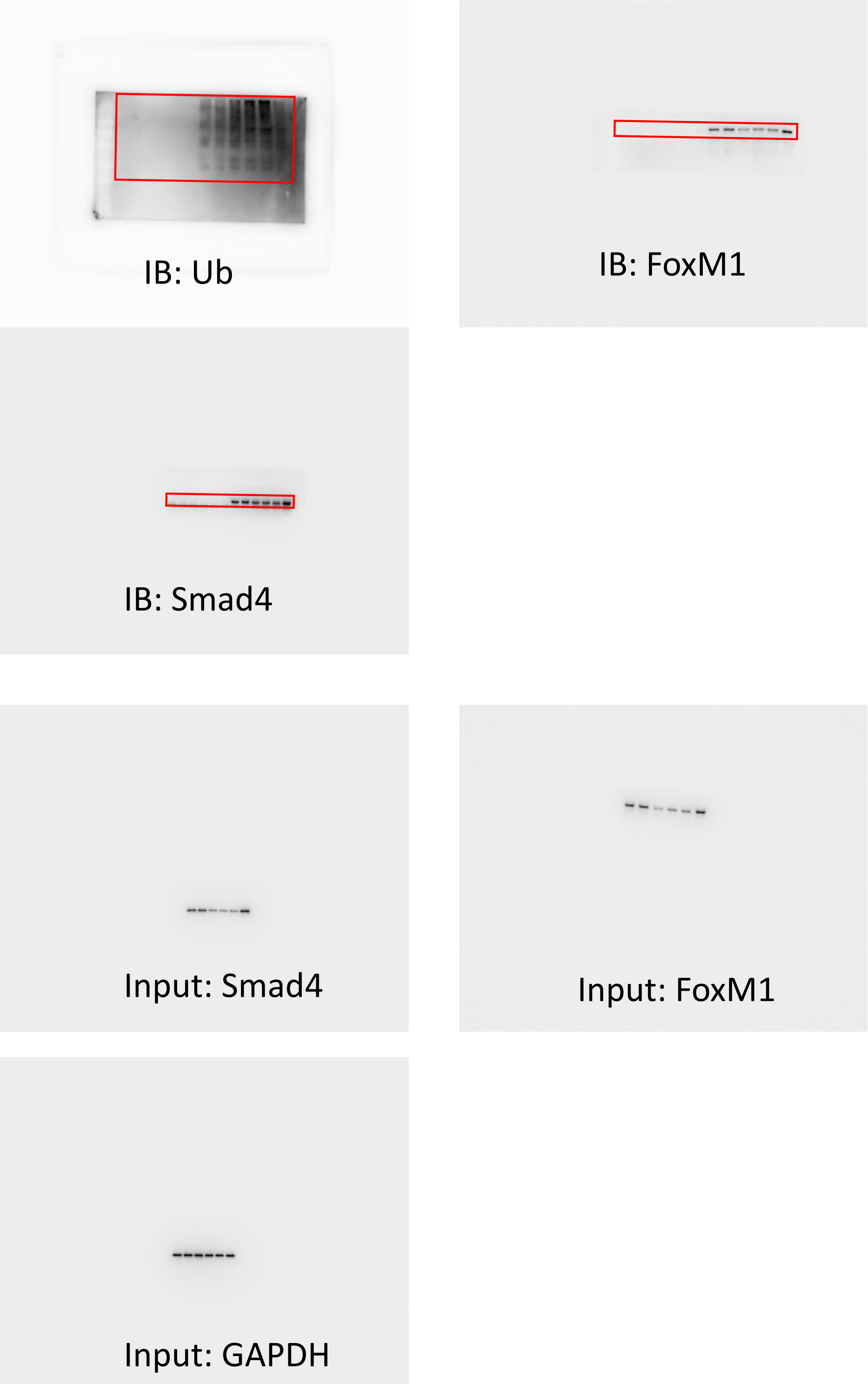

Figure 5A

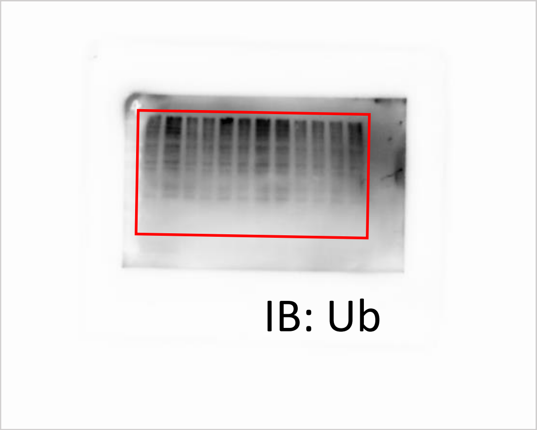

IB: Ub

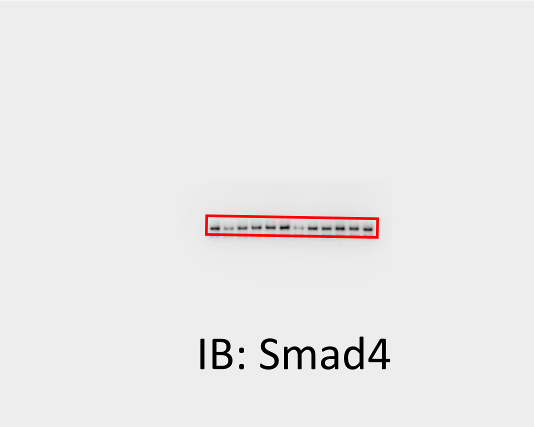

IB: Smad4

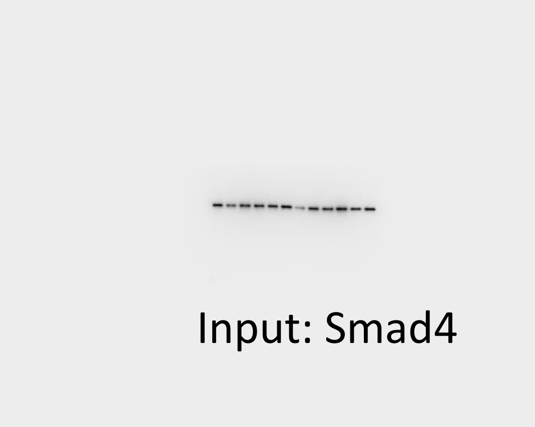

Input: Smad4

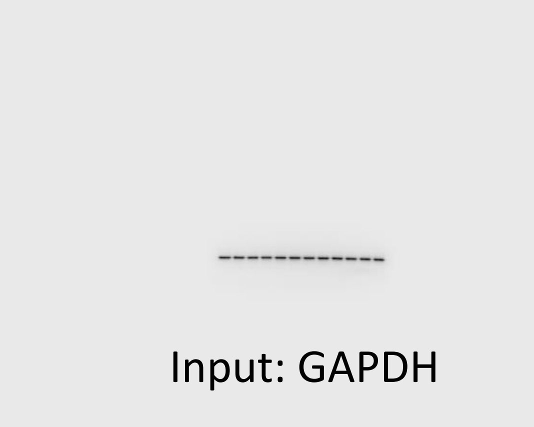

Input: GAPDH

Figure 5C

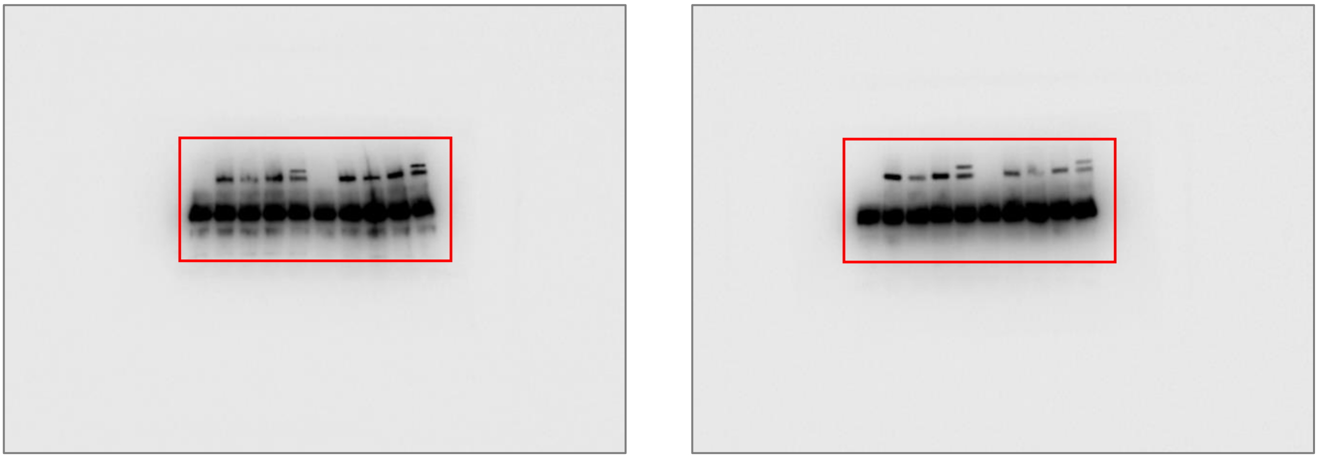

Figure 6C

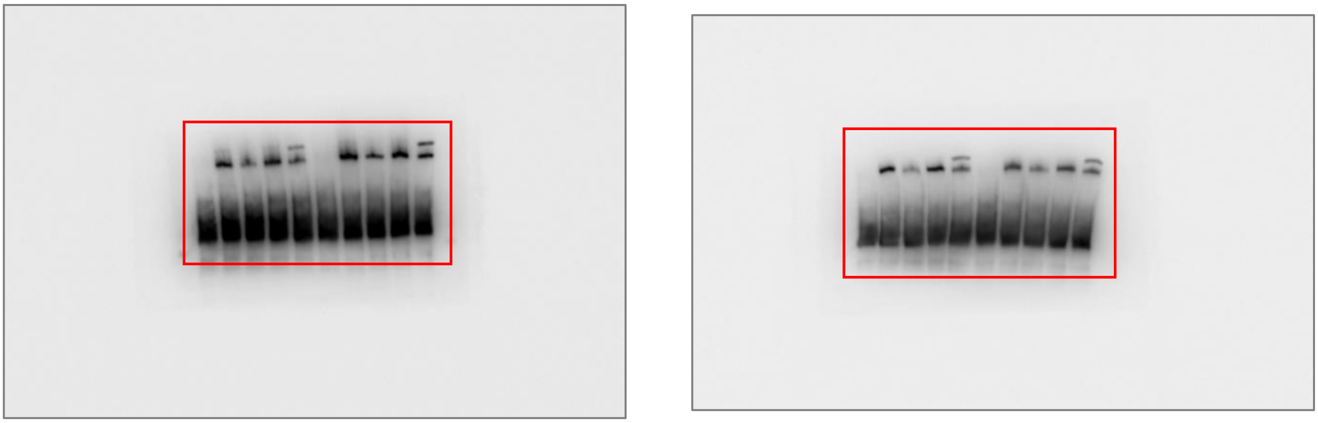

Figure 6D

Fig. S2B

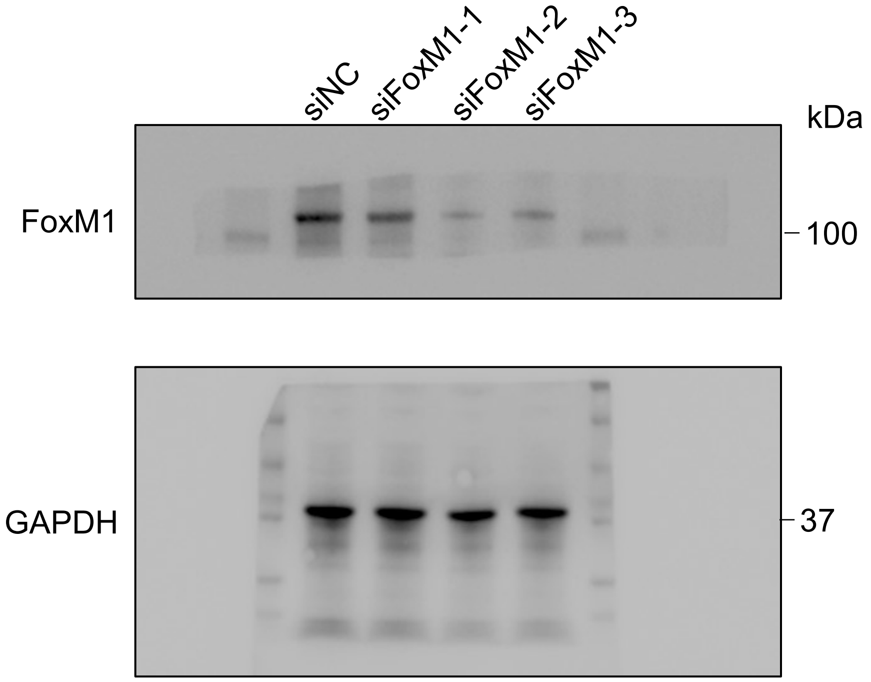

Fig. S4C

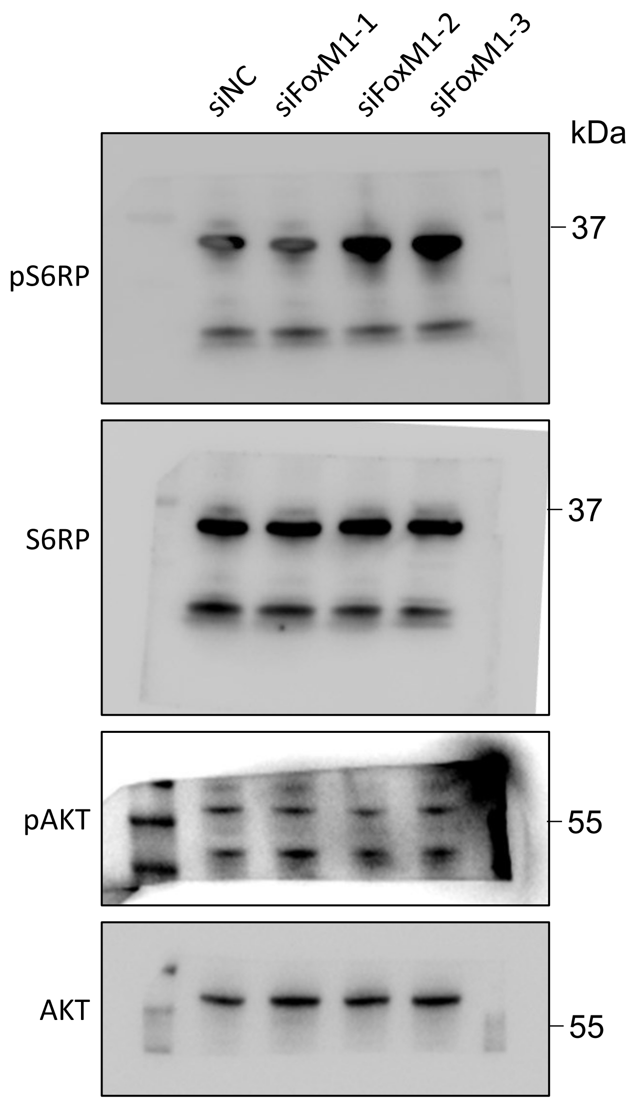
siNC
siFoxM1-1
siFoxM1-2
siFoxM1-3
kDa
pS6RP
37
S6RP
37
pAKT
55
AKT
55

备注：实时荧光实验结果采用2-ΔΔCT的数据统计方法

9.559662882

Sample	Target Name	Task	Reporter	Quencher	Cт	GAPDH mean（ct）	ΔCT	ΔΔCT	负ΔΔCT	2-ΔΔCT	2-ΔΔCT M	Ct SD			
HPDE6-C7	FoxM1	UNKNOWN	SYBR	None	28.14163361		9.683700816	0.124038	-0.12404	0.917616	1.001841	0.073351	1	0.07 ±	1±0.07
	FoxM1	UNKNOWN	SYBR	None	27.94487915		9.486946360	-0.07272	0.072717	1.051695					
	FoxM1	UNKNOWN	SYBR	None	27.96627426		9.508341471	-0.05132	0.051321	1.036214					
	GAPDH	UNKNOWN	SYBR	None	18.37805634	18.45793279									
	GAPDH	UNKNOWN	SYBR	None	18.5104126										
	GAPDH	UNKNOWN	SYBR	None	18.48532944										
PANC-1	FoxM1	UNKNOWN	SYBR	None	25.29553452		6.490357463	-3.06931	3.069305	8.393691	8.426857	0.107293	8.43	0.11 ±	8.43±0.11
	FoxM1	UNKNOWN	SYBR	None	25.30478172		6.499604670	-3.06006	3.060058	8.340063					
	FoxM1	UNKNOWN	SYBR	None	25.26945267		6.464275614	-3.09539	3.095387	8.546817					
	GAPDH	UNKNOWN	SYBR	None	18.81337242	18.80517705									
	GAPDH	UNKNOWN	SYBR	None	18.81122208										
	GAPDH	UNKNOWN	SYBR	None	18.79093666										
SW1990	FoxM1	UNKNOWN	SYBR	None	22.51769447		6.975727717	-2.58394	2.583935	5.995729	5.555057	0.381788	5.56	0.38 ±	5.56±0.38
	FoxM1	UNKNOWN	SYBR	None	22.6891655		7.147198741	-2.41246	2.412464	5.323829					
	FoxM1	UNKNOWN	SYBR	None	22.68327408		7.141307322	-2.41836	2.418356	5.345614					
	GAPDH	UNKNOWN	SYBR	None	15.67357903	15.54196676									
	GAPDH	UNKNOWN	SYBR	None	15.40384121										
	GAPDH	UNKNOWN	SYBR	None	15.54848003										
Panc10.05	FoxM1	UNKNOWN	SYBR	None	33.40637054		8.832446162	-0.72722	0.727217	1.655442	1.678056	0.029112	1.68	0.03 ±	1.68±0.03
	FoxM1	UNKNOWN	SYBR	None	33.35882874		8.784904353	-0.77476	0.774759	1.710904					
	FoxM1	UNKNOWN	SYBR	None	33.39562149		8.821697108	-0.73797	0.737966	1.667823					
	GAPDH	UNKNOWN	SYBR	None	24.47338295	24.57392438									
	GAPDH	UNKNOWN	SYBR	None	24.56637192										
	GAPDH	UNKNOWN	SYBR	None	24.68201828										
CFPAC-1	FoxM1	UNKNOWN	SYBR	None	28.17457657		7.342371114	-2.21729	2.217292	4.650197	4.556555	0.249085	4.56	0.25 ±	4.56±0.25
	FoxM1	UNKNOWN	SYBR	None	28.14538498		7.313179525	-2.24648	2.246483	4.745248					
	FoxM1	UNKNOWN	SYBR	None	28.29620743		7.464001973	-2.09566	2.095661	4.274219					
	GAPDH	UNKNOWN	SYBR	None	20.86076241	20.83220545									
	GAPDH	UNKNOWN	SYBR	None	20.84953346										
	GAPDH	UNKNOWN	SYBR	None	20.7863205										
PATU8988	FoxM1	UNKNOWN	SYBR	None	26.12724953		6.838357544	-2.72131	2.721305	6.594692	6.179317	0.360966	6.18	0.36 ±	6.18±0.36
	FoxM1	UNKNOWN	SYBR	None	26.27767143		6.988779449	-2.57088	2.570883	5.941732					
	FoxM1	UNKNOWN	SYBR	None	26.26322517		6.974333191	-2.58533	2.58533	6.001527					
	GAPDH	UNKNOWN	SYBR	None	19.30038681	19.28889198									
	GAPDH	UNKNOWN	SYBR	None	19.28292923										
	GAPDH	UNKNOWN	SYBR	None	19.28335991										
MIA-PACA-2	FoxM1	UNKNOWN	SYBR	None	26.23025551		7.900284831	-1.65938	1.659378	3.158803	3.479734	0.297144	3.48	0.3 ±	3.48±0.3
	FoxM1	UNKNOWN	SYBR	None	25.98455086		7.654580180	-1.90508	1.905083	3.745304					
	FoxM1	UNKNOWN	SYBR	None	26.06788483		7.737914149	-1.82175	1.821749	3.535094					
	GAPDH	UNKNOWN	SYBR	None	18.35634651	18.32997068									
	GAPDH	UNKNOWN	SYBR	None	18.32736664										
	GAPDH	UNKNOWN	SYBR	None	18.30619888										
capan-1	FoxM1	UNKNOWN	SYBR	None	34.22123718		11.942988586	2.383326	-2.38333	0.191667	0.205849	0.013253	0.21	0.01 ±	0.21±0.01
	FoxM1	UNKNOWN	SYBR	None	34.03604507		11.757796478	2.198134	-2.19813	0.217919					
	FoxM1	UNKNOWN	SYBR	None	34.10353088		11.825282288	2.265619	-2.26562	0.20796					
	GAPDH	UNKNOWN	SYBR	None	22.23952065	22.2782486									
	GAPDH	UNKNOWN	SYBR	None	22.28032875										
	GAPDH	UNKNOWN	SYBR	None	22.31489639										
capan-2	FoxM1	UNKNOWN	SYBR	None	27.04229088		7.582834117	-1.97683	1.976829	3.936269	3.725601	0.228622	3.73	0.23 ±	3.73±0.23
	FoxM1	UNKNOWN	SYBR	None	27.21899986		7.759543101	-1.80012	1.80012	3.482491					
	FoxM1	UNKNOWN	SYBR	None	27.10913811		7.649681346	-1.90998	1.909982	3.758043					
	GAPDH	UNKNOWN	SYBR	None	19.35461235	19.45945676									
	GAPDH	UNKNOWN	SYBR	None	19.53730392										
	GAPDH	UNKNOWN	SYBR	None	19.48645401										
AsPC-1	FoxM1	UNKNOWN	SYBR	None	36.62849426		13.030616124	3.470953	-3.47095	0.090186	0.091034	0.002286	0.09	0 ±	0.09±0
	FoxM1	UNKNOWN	SYBR	None	36.64285278		13.044974645	3.485312	-3.48531	0.089293					
	FoxM1	UNKNOWN	SYBR	None	36.57454376		12.976665624	3.417003	-3.417	0.093622					
	GAPDH	UNKNOWN	SYBR	None	23.62933197	23.59787814									
	GAPDH	UNKNOWN	SYBR	None	23.5489933										
	GAPDH	UNKNOWN	SYBR	None	23.61530914										

group	FoxM1
HPDE6-C7	1±0.07
PANC-1	8.43±0.11
SW1990	5.56±0.38
Panc10.05	1.68±0.03
CFPAC-1	4.56±0.25
PATU8988	6.18±0.36
MIA-PACA-2	3.48±0.3
capan-1	0.21±0.01
capan-2	3.73±0.23
AsPC-1	0.09±0

Ave	Std
1	0.07
8.43	0.11
5.56	0.38
1.68	0.03
4.56	0.25
6.18	0.36
3.48	0.30
0.21	0.01
3.73	0.23
0.09	0.00

备注：实时荧光实验结果采用2-ΔΔCT的数据统计方法							10.67700526									
Sample	Target Name	Task	Reporter	Quencher	Cт	GAPDH mean（ct）	ΔCT	ΔΔCT	负ΔΔCT	2-ΔΔCT	2-ΔΔCT M	Ct SD				
Panc10.05	FoxM1	UNKNOWN	SYBR	None	27.87528267		10.597753270	-0.079251989	0.07925	1.05647	1.00093	0.05302		1	0.05 ±	1±0.05
	FoxM1	UNKNOWN	SYBR	None	27.96108627		10.683556875	0.006551615	-0.00655	0.99547						
	FoxM1	UNKNOWN	SYBR	None	28.02723503		10.749705633	0.072700373	-0.0727	0.95086						
	GAPDH	UNKNOWN	SYBR	None	17.25727425	17.2775294										
	GAPDH	UNKNOWN	SYBR	None	17.32572441											
	GAPDH	UNKNOWN	SYBR	None	17.24958954											
Panc10.05+过表达NC	FoxM1	UNKNOWN	SYBR	None	31.89176514		10.701632996	0.024627736	-0.02463	0.98307	0.97328	0.03752		0.97	0.04 ±	0.97±0.04
	FoxM1	UNKNOWN	SYBR	None	31.96899109		10.778858948	0.101853689	-0.10185	0.93183						
	FoxM1	UNKNOWN	SYBR	None	31.86005325		10.669921112	-0.007084147	0.00708	1.00492						
	GAPDH	UNKNOWN	SYBR	None	21.13750267	21.19013214										
	GAPDH	UNKNOWN	SYBR	None	21.29050064											
	GAPDH	UNKNOWN	SYBR	None	21.14239311											
Panc10.05+过表达	FoxM1	UNKNOWN	SYBR	None	21.55816269		1.818272909	-8.858732351	8.85873	464.242	465.64	21.564	3.0774E-06	465.64	21.56 ±	465.64±21.56
	FoxM1	UNKNOWN	SYBR	None	21.48654289		1.746653112	-8.930352147	8.93035	487.87						
	FoxM1	UNKNOWN	SYBR	None	21.61985016		1.879960378	-8.797044881	8.79704	444.81						
	GAPDH	UNKNOWN	SYBR	None	19.75215149	19.73988978										
	GAPDH	UNKNOWN	SYBR	None	19.71606827											
	GAPDH	UNKNOWN	SYBR	None	19.75144958		6.886909612									
Patu8988	FoxM1	UNKNOWN	SYBR	None	24.23426247		6.925562541	0.038652928670277	-0.03865	0.97356	1.00028	0.02911		1	0.03 ±	1±0.03
	FoxM1	UNKNOWN	SYBR	None	24.20141716		6.892717234	0.005807622	-0.00581	0.99598						
	FoxM1	UNKNOWN	SYBR	None	24.15114899		6.842449061	-0.044460551	0.04446	1.0313						
	GAPDH	UNKNOWN	SYBR	None	17.2410965	17.30869993										
	GAPDH	UNKNOWN	SYBR	None	17.30162315											
	GAPDH	UNKNOWN	SYBR	None	17.38338013											
Patu8988+干扰NC	FoxM1	UNKNOWN	SYBR	None	25.41261749		6.965349325	0.078439713	-0.07844	0.94708	0.98783	0.04229		0.99	0.04 ±	0.99±0.04
	FoxM1	UNKNOWN	SYBR	None	25.35612411		6.908855947	0.021946335	-0.02195	0.9849						
	FoxM1	UNKNOWN	SYBR	None	25.28941269		6.842144521	-0.044765091	0.04477	1.03152						
	GAPDH	UNKNOWN	SYBR	None	18.44805946	18.44726817										
	GAPDH	UNKNOWN	SYBR	None	18.37006721											
	GAPDH	UNKNOWN	SYBR	None	18.52367783											
Patu8988+干扰-1	FoxM1	UNKNOWN	SYBR	None	34.64835434		6.802063751	-0.084845861	0.08485	1.06057	1.06055	0.05781	0.153488971	1.06	0.06 ±	1.06±0.06
	FoxM1	UNKNOWN	SYBR	None	34.7292598		6.882969208	-0.003940404	0.00394	1.00274						
	FoxM1	UNKNOWN	SYBR	None	34.57182693		6.725536346	-0.161373266	0.16137	1.11835						
	GAPDH	UNKNOWN	SYBR	None	27.8051815	27.84629059										
	GAPDH	UNKNOWN	SYBR	None	27.79105568											
	GAPDH	UNKNOWN	SYBR	None	27.94263458											
Patu8988+干扰-2	FoxM1	UNKNOWN	SYBR	None	26.3833168		7.821931330	0.935021718	-0.93502	0.52303	0.53481	0.03244	0.000123906	0.53	0.03 ±	0.53±0.03
	FoxM1	UNKNOWN	SYBR	None	26.25548553		7.694100062	0.80719045	-0.80719	0.57149						
	FoxM1	UNKNOWN	SYBR	None	26.41997528		7.858589808	0.971680196	-0.97168	0.50991						
	GAPDH	UNKNOWN	SYBR	None	18.4791729	18.56138547										
	GAPDH	UNKNOWN	SYBR	None	18.52721863											
	GAPDH	UNKNOWN	SYBR	None	18.67776489											
Patu8988+干扰-3	FoxM1	UNKNOWN	SYBR	None	27.43348045		8.658721161	1.771811549	-1.77181	0.29284	0.28995	0.01984	1.32551E-05	0.29	0.02 ±	0.29±0.02
	FoxM1	UNKNOWN	SYBR	None	27.55695381		8.782194519	1.895284907	-1.89528	0.26882						
	FoxM1	UNKNOWN	SYBR	None	27.35976448		8.585005188	1.698095576	-1.6981	0.30819						
	GAPDH	UNKNOWN	SYBR	None	18.75303993	18.77475929										
	GAPDH	UNKNOWN	SYBR	None	18.70973969											
	GAPDH	UNKNOWN	SYBR	None	18.86149826								0.153488971			
													0.000123906			
													1.32551E-05			

group	FoxM1	Ave	STD
Panc10.05	1±0.05	1	0.05
Panc10.05+过表达NC	0.97±0.04	0.97	0.04
Panc10.05+过表达	465.64±21.56	465.64	21.56
Patu8988	1±0.03	1	0.03
Patu8988+干扰NC	0.99±0.04	0.99	0.04
Patu8988+干扰-1	1.06±0.06	1.06	0.06
Patu8988+干扰-2	0.53±0.03	0.53	0.03
Patu8988+干扰-3	0.29±0.02	0.29	0.02

Ct	Ct平均值	Ct SD

孔	样品名称	样品类型	染料	基因	Ct	Ct平均值	Ct SD
A1	NC	未知样品	FAM	FOXM1	18.01	18.15	0.35
A2	NC	未知样品	FAM	FOXM1	17.88	18.15	0.35
A3	NC	未知样品	FAM	FOXM1	18.55	18.15	0.35
A4	SI1	未知样品	FAM	FOXM1	24.39	24.5	0.29
A5	SI1	未知样品	FAM	FOXM1	24.29	24.5	0.29
A6	SI1	未知样品	FAM	FOXM1	24.83	24.5	0.29
A7	SI2	未知样品	FAM	FOXM1	24.13	23.87	0.23
A8	SI2	未知样品	FAM	FOXM1	23.74	23.87	0.23
A9	SI2	未知样品	FAM	FOXM1	23.74	23.87	0.23
A10	SI3	未知样品	FAM	FOXM1	24.14	24.3	0.26
A11	SI3	未知样品	FAM	FOXM1	24.61	24.3	0.26
A12	SI3	未知样品	FAM	FOXM1	24.16	24.3	0.26
B1	NC	未知样品	FAM	GADPH	11.86	11.89	0.35
B2	NC	未知样品	FAM	GADPH	12.26	11.89	0.35
B3	NC	未知样品	FAM	GADPH	11.56	11.89	0.35
B4	SI1	未知样品	FAM	GADPH	13.32	13.3	0.03
B5	SI1	未知样品	FAM	GADPH	13.27	13.3	0.03
B6	SI1	未知样品	FAM	GADPH	13.31	13.3	0.03
B7	SI2	未知样品	FAM	GADPH	12.46	12.68	0.2
B8	SI2	未知样品	FAM	GADPH	12.85	12.68	0.2
B9	SI2	未知样品	FAM	GADPH	12.75	12.68	0.2
B10	SI3	未知样品	FAM	GADPH	12.29	12.51	0.46
B11	SI3	未知样品	FAM	GADPH	13.04	12.51	0.46
B12	SI3	未知样品	FAM	GADPH	12.19	12.51	0.46

6.2533333

目标基因	Cq	ct 18s	d CT	dd CT	POWER	AVERAGE	STD
NC	18.01	11.86	6.1166667	-0.136667	1.0993621	1.01949881	0.2339167
NC	17.88	12.26	5.9866667	-0.266667	1.203025		
NC	18.55	11.56	6.6566667	0.4033333	0.7561093		
SI1	24.39	13.32	11.09	4.8366667	0.034996	0.032766837	0.0061655
SI1	24.29	13.27	10.99	4.7366667	0.0375078		
SI1	24.83	13.31	11.53	5.2766667	0.0257968		
SI2	24.13	12.46	11.443333	5.19	0.0273939	0.033062525	0.0049091
SI2	23.74	12.85	11.053333	4.8	0.0358968		
SI2	23.74	12.75	11.053333	4.8	0.0358968		
SI3	24.14	12.29	11.633333	5.38	0.0240137	0.021677923	0.003763
SI3	24.61	13.04	12.103333	5.85	0.017337		
SI3	24.16	12.19	11.653333	5.4	0.0236831		
